# Inborn errors of immunity underlie clonal T cell expansions in large granular lymphocyte leukemia

**DOI:** 10.1172/JCI184431

**Published:** 2025-05-01

**Authors:** Carlos Bravo-Perez, Carmelo Gurnari, Jani Huuhtanen, Naomi Kawashima, Luca Guarnera, Aashray Mandala, Nakisha D. Williams, Christopher Haddad, Michaela Witt, Serhan Unlu, Zachary Brady, Olisaemeka Ogbue, Mark Orland, Arooj Ahmed, Yasuo Kubota, Simona Pagliuca, Arda Durmaz, Satu Mustjoki, Valeria Visconte, Jaroslaw P. Maciejewski

**Affiliations:** 1 Department of Translational Hematology and Oncology Research, Taussig Cancer Institute, Cleveland Clinic, Cleveland, Ohio, USA.; 2 Department of Hematology and Medical Oncology, Hospital Universitario Morales Meseguer, University of Murcia, IMIB-Pascual Parrilla, CIBERER–Instituto de Salud Carlos III, Murcia, Spain.; 3 Department of Biomedicine and Prevention, University of Rome Tor Vergata, Rome, Italy.; 4 Hematology Research Unit Helsinki, Department of Hematology, University of Helsinki and Helsinki University Hospital Comprehensive Cancer Center, Helsinki, Finland.; 5 Translational Immunology Research Program, University of Helsinki, Helsinki, Finland.; 6 ICAN Digital Precision Cancer Medicine Flagship, University of Helsinki and Helsinki University Hospital Comprehensive Cancer Center, Helsinki, Finland.; 7 Department of Computer Science, Aalto University, Espoo, Finland.; 8 Hematology Department, Nancy University Hospital and UMR 7365 CNRS University of Lorraine, France.

**Keywords:** Genetics, Hematology, Adaptive immunity, Lymphomas, Molecular genetics

## Abstract

**BACKGROUND:**

T cell large granular lymphocyte leukemia (T-LGLL) is a lymphoproliferative disorder of cytotoxic T lymphocytes (CTLs), often with gain-of-function *STAT3* mutations. T-LGLL represents a unique model for the study of persistent CTL expansions. Albeit autoimmunity is implied, various paradoxical observations led us to investigate whether immunodeficiency traits underpin T-LGLL.

**METHODS:**

This is a comprehensive immunogenomic study of 92 consecutive patients from a large T-LGLL cohort with full laboratory-clinical characterization (*n* = 271). Whole-exome profiling of variants associated with inborn errors of immunity (IEI) and somatic mutations in T cell lymphoid drivers was analyzed. Single-cell RNA-Seq and TCR-Seq in T-LGLL samples and RNA-Seq in T cell cancer cell lines were utilized to establish biological correlations.

**RESULTS:**

Lymphocytopenia and/or hypogammaglobulinemia were identified in 186 of 241 (77%) T-LGLL patients. Genetic screening for IEI revealed 43 rare heterozygous variants in 38 different immune genes in 34 of 92 (36%) patients (vs. 167/63,026 [0.26%] in controls). High-confidence deleterious variants associated with dominant, adult-onset IEIs were detected in 15 of 92 (16%) patients. Carriers showed atypical features otherwise tied to the cryptic IEI, such as earlier onset, lower lymphocyte counts, lower *STAT3* mutational rate, and higher proportions of hypogammaglobulinemia and immune cytopenia/bone marrow failure than noncarriers. Somatic mutational landscape, RNA-Seq, and TCR-Seq analyses supported immune imbalance caused by the IEI variants and interactions with somatic mutations in T cell lymphoid drivers.

**CONCLUSIONS:**

Our findings in T-LGLL reveal that maladaptive CTL expansions may stem from cryptic immunodeficiency traits and open the horizon of IEIs to clonal hematopoiesis and bone marrow failure.

**FUNDING:**

NIH; Aplastic Anemia and MDS International Foundation; VeloSano; Edward P. Evans Foundation; Instituto de Salud Carlos III; European Research Council; European Research Area Network on Personalised Medicine; Academy Finland; Cancer Foundation Finland.

## Abstract 2

This study in T-LGLL reveals that maladaptive CTL expansions may stem from cryptic immunodeficiency traits, and open the horizon of IEIs to clonal hematopoiesis and bone marrow failure.

## Graphical abstract

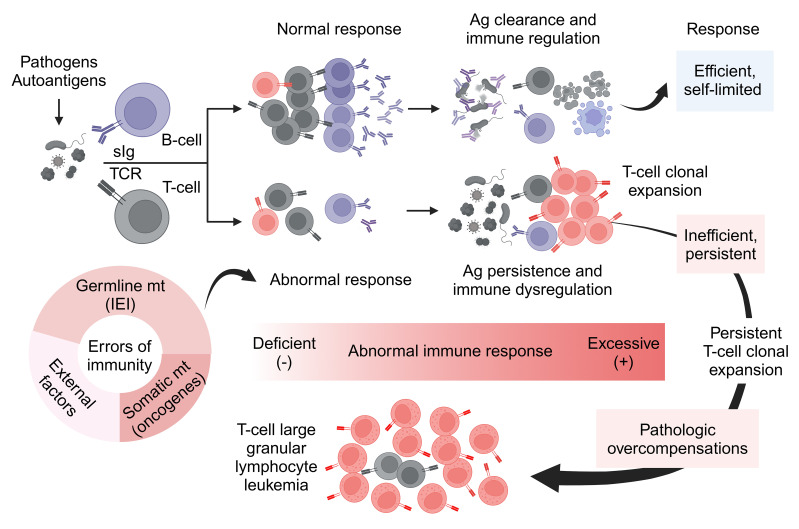

## Introduction

T cell large granular lymphocyte leukemia (T-LGLL) is a chronic lymphoproliferative disorder of cytotoxic T lymphocytes (CTLs) (1). Common in the elderly, the disease spans a continuum from reactive poly/oligoclonal responses to clonally skewed CTLs. During its natural course, patients eventually develop severe cytopenias or paraneoplastic autoimmune complications requiring therapy (2). Gain-of-function (GOF) *STAT3* mutations (*STAT3mt*) occur in 40%–50% of T-LGLL patients and normally imply a more severe clinical phenotype. However, T cell large granular lymphocyte (T-LGL) leukemic clones normally show constant JAK/STAT activation irrespective of the *STAT3*mt status. Clinical and laboratory advances have also revealed the dysregulation of alternative survival pathways in patients with *STAT3* wild-type (*STAT3*wt) T-LGLL (e.g., MCL1, NF-κB, PI3K/AKT) (3–6). Mutations in driver genes other than *STAT3*, recurrent in mature T cell neoplasms of other subtypes, have been reported, such as in *STAT5B*, *TNFAIP3*, and the epigenetic regulators *KMT2D* or *TET2* (7).

The precise pathogenesis of T-LGLL remains ill defined. However, its cooccurrence with different autoimmune, neoplastic, or infectious conditions is well known (8). Chronic antigenic stimulation has been hypothesized to initiate/drive CTL outgrowth in T-LGLL mostly based on these supporting clinical associations. More recently, laboratory evidence suggests the role of the immune microenvironment and its crosstalk with the T-LGL clone via elevated levels of proinflammatory cytokines (e.g., IL-15, TNF-α, IL-6, IFN-γ) (9, 10). For instance, high levels of IL-15 induce T-LGL lymphoproliferative disease in transgenic mice (10, 11), and blockade with anti–IL-15 in clinical trials has shown partial restoration of the resistance of T-LGL cells to apoptosis (12). Accordingly, T-LGLL appears to correspond with an overshooting immune reaction in which immunodominant clones, cumulatively fueled by chronic (auto)antigen exposure, and environmental and/or genetic growth signals may directly act as effector CTLs or cause cytokine-mediated manifestations (13, 14). Thus, despite being extremely rare, T-LGLL represents a unique disease model, at the intersection of a physiological immune response, autoimmunity, and malignancy.

Our ongoing study of clinical features of T-LGLL suggests that, despite indications of a hyperreactive immune response, a paradoxically high frequency of immunodeficiency signs might be present at diagnosis. For instance, despite fulfilling the diagnostic criteria, the disease is not always characterized by lymphocytosis as expected from a frank leukemia, but may be associated with relatively low/normal lymphocyte counts. Moreover, hypogammaglobulinemia is also frequently encountered. We previously described hypogammaglobulinemia in T-LGLL among patients with a history of B cell dyscrasia or transplant (15, 16). We also demonstrated obvious or occult T-LGLL as a novel feature of Good syndrome (17). In patients with common variable immunodeficiency (CVID), a high rate of somatic mutations and clonality in T cells has been reported, suggesting a propensity to clonal outgrowth (18).

While admittedly development of T-LGLL is a complex process, the initial overshooting immune response might be specifically triggered in genetically predisposed subjects. We hypothesized that certain errors of immunity may induce maladaptive CTL responses, ultimately leading to the clonal shift. In this context, we aimed to systematically assess the acquired and genetic immunodeficiency underpinnings of T-LGLL.

## Results

### Clinical dissection of immune defects in a large T-LGLL cohort.

**Figure 1:**
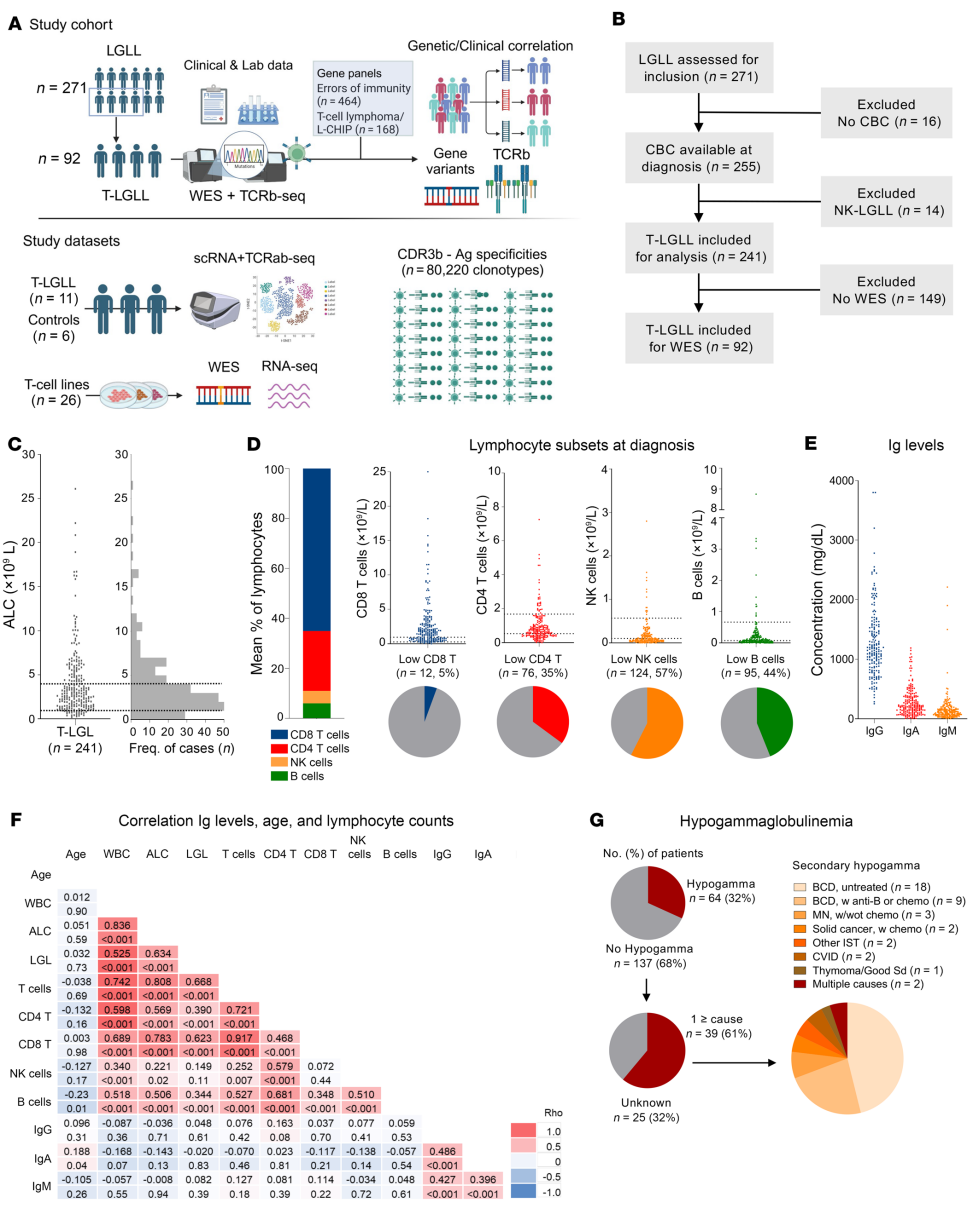
Study summary and systematic characterization of immunodeficiency underpinnings of T-LGLL. (**A**) Graphic summary of study design, study cohort, and study datasets (created with Biorender.com). (**B**) Flow chart of the initial immunologic laboratory characterization. (**C**) Distribution dot plot of cohort absolute lymphocyte count (ALC) (available in *n* = 241 patients). Dashed lines represent the lower and upper limits of the normal range. The histogram represents the number of cases within ALC ranges. (**D**) Lymphocyte populations in peripheral blood at diagnosis (available in *n* = 216 patients). The bar chart represents the mean percentages of lymphocyte populations (CD8^+^ T cells, CD4^+^ T cells, NK and B cells). Distribution dot plots of each lymphocyte subset are also shown. Dashed lines represent the lower and upper limits of the normal range. The pie charts highlight the cases with low CD8^+^ T cells, low CD4^+^ T cells, low NK cells, and low B cells. (**E**) Concentration of Ig isotypes (IgG, IgA, IgM) at diagnosis (available in *n* = 201 patients). (**F**) Correlogram showing the association of Ig levels with age, blood counts, and lymphocytes. Spearman’s rho correlation coefficients and *P* values are shown for each pair of variables. Color scale represents the magnitude of the correlation coefficient. (**G**) Screening for potential causes of Ig hypogammaglobulinemia, detected in *n* = 64 patients at diagnosis. Anti-B, anti–B cell therapy; BCD, B cell dyscrasia; CBC, complete blood count; Chemo, chemotherapy; IST, immunosuppressive therapy; LGL, large granular lymphocyte count; WBC, white blood cell count; MN, myeloid neoplasms.

The study was conducted on a cohort of 271 consecutive T-LGLL patients with full annotations diagnosed at Cleveland Clinic (1998-2023; Figure 1A and Table 1). Because of our focus on T cell clonal expansions, cases with NK-cell LGLL subtype were excluded. The initial analysis of clinical features confirmed a high frequency of different immune defects at T-LGLL diagnosis (186 [77%] of 241 patients). Of 241 cases with blood counts at presentation, 169 (66%) had low/normal absolute lymphocyte counts (≤4.0 × 10^9^/L; Figure 1, B and C). Blood lymphocyte subset immunophenotyping in 216 patients revealed reduced levels of CD4^+^ T cells in 76 (35%), low NK cells in 124 (58%), and low B cells in 95 (44%) cases (Figure 1D). In addition, 64 (32%) of 201 patients with Ig levels had hypogammaglobulinemia, including 29 (14%), 32 (16%), and 36 (18%) of 201 patients with low IgG, IgA, and IgM, respectively (Figure 1, E and F). Clinical history indicated that 39 (61%) of 64 cases with hypogammaglobulinemia could have been acquired, given the prior diagnosis of hemato-lymphoid neoplasms, anti–B cell or other immunosuppressive treatments, and Good syndrome or related CVID-like conditions. However, in 25 (39%) of 64 hypogammaglobulinemic patients, no apparent factors for secondary deficiency were identified (Figure 1G).

### Immunogenomic profiling of rare variants predisposing to errors of immunity.

Focusing on the investigation of genetic immunodeficiency traits as predisposing factors for T-LGLL, we subjected a representative subcohort of 92 consecutive patients (38% of the whole cohort, no clinically biased criteria applied) to whole-exome sequencing (WES) (Table 1). The median age at diagnosis was 62 years (IQR: 54–71) with a male:female ratio of 0.5. Mutations in *STAT3* and *STAT5B* were present in 42 (46%) and 1 (1%) of 92 patients, respectively. We then analyzed the open reading frames of 464 genes associated with inborn errors of immunity (IEI) included in the 2022 classification of the International Union of Immunological Societies (IUIS; Supplemental Table 1; supplemental material available online with this article; https://doi.org/10.1172/JCI184431DS1) (19). Rare variants were defined by minor allele frequencies (MAF) in the Genome Aggregation Database (gnomAD) of less than 1%. Each variant was assessed according to American College of Medical Genetics (ACMG) criteria using ClinVar (20) and VarSome (21). For analysis, we selected variants classified as pathogenic/likely pathogenic (P/LP), or variants of uncertain significance (VUS) overrepresented in our cohort, i.e., those with a significant *P* value for observed versus expected frequencies after Benjamini-Hochberg multiple-testing correction (FDR < 0.05).

**Figure 2:**
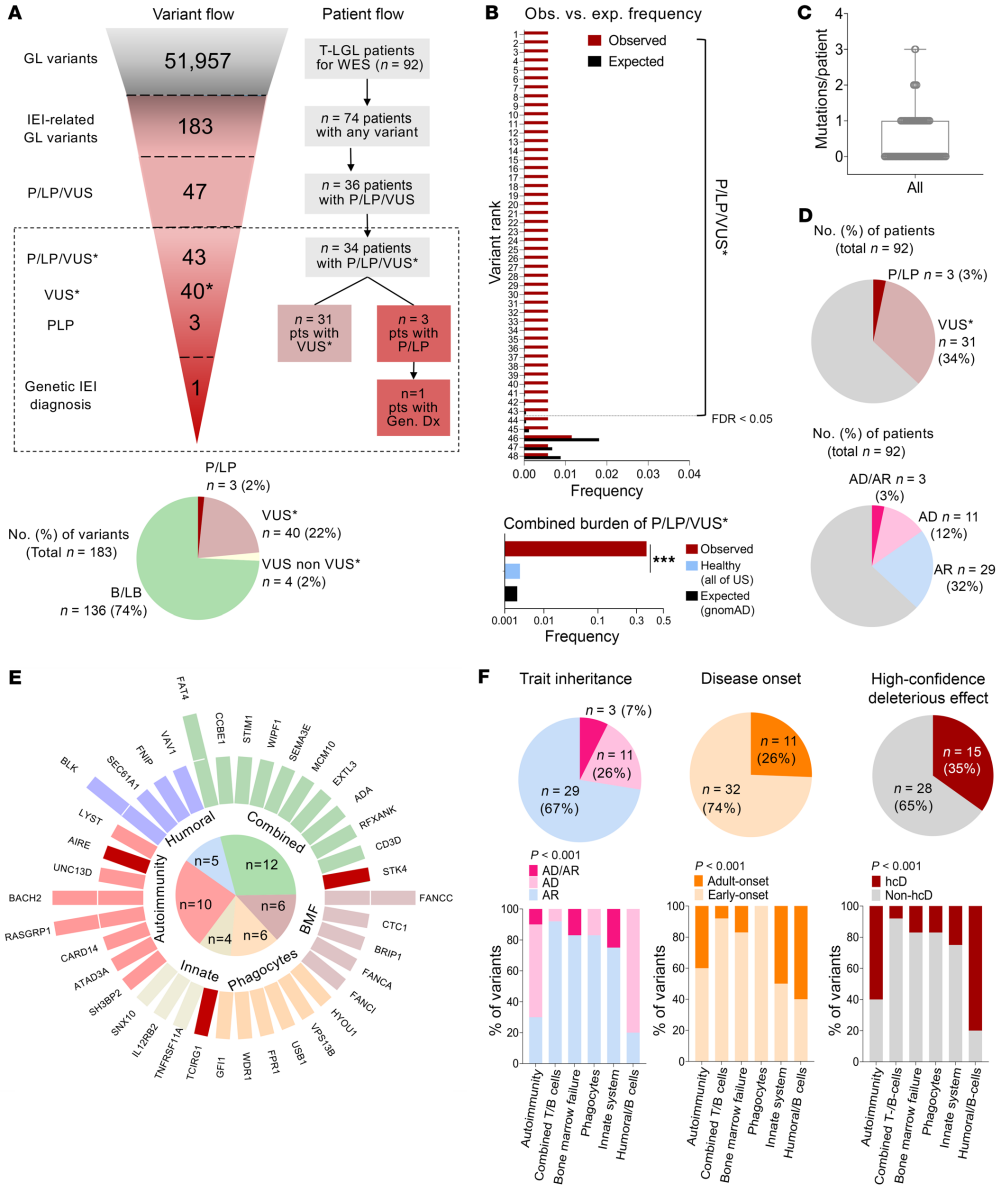
Immunogenomic landscape of variants predisposing to IEI in patients with T-LGLL. (**A**) Variant and patient flow. Variants of potential clinical relevance included in the analysis (P/LP/VUS*) are highlighted with a dashed box. The pie chart at the bottom indicates the distribution of the variants by pathogenicity (ACMG criteria). (**B**) Comparison of the observed (Obs.) versus expected (exp.) (gnomAD) allele frequencies for the selection of variants overrepresented in our cohort (P/LP/VUS*). Multiple testing correction was done with Benjamini-Hodgberg method (FDR < 0.05). The bar chart at the bottom represents the combined mutational burden of all P/LP/VUS* observed in our cohort (39% vs. 0.26%, as compared with *All of US* healthy controls with genomic data, *n* = 63,026). χ^2^ *P* values are shown. The expected probability of finding any of the variants in the general population according to gnomAD is also shown (0.22%). (**C**) Individual mutation burden among carriers. All variants were found in the heterozygous state. (**D**) Number of patients carrying the variants according to pathogenicity (upper chart) and pattern of inheritance (lower chart). (**E**) Circle plot representing the number of variants by gene according to functional clustering and pathogenicity. Maroon bars indicate P/LP and genetic diagnoses of IEI, respectively. (**F**) Proportion of variants found according to functionality and pathogenicity, trait inheritance, and disease onset. χ^2^ *P* values are shown. ***P < 0.001. AD, autosomal dominant; AR, autosomal recessive; B/LB, benign/likely benign; Gen. Dx, genetic diagnosis; GL, germline; VUS*, VUS overrepresented in our cohort.

We found 183 suspicious rare germline variants in IEI-related genes (Figure 2A). Of those, we selected 43 variants of potential clinical relevance (40 VUS; 3 P/LP) in 38 different genes that were overrepresented in our cohort after comparing observed versus expected allele frequencies in the general population. These variants were detected in 34 (37%) of 92 patients, a combined genetic burden significantly elevated when compared with the *All* *of US* (https://allofus.nih.gov/) healthy control population with genomic data, of whom only 167 (0.26%) of 63,026 individuals carried any of the variants identified (*P* < 0.001; Figure 2B and Supplemental Table 2). The median number of variants in carriers was 1 (range: 1–3 mutations/patient). All variants were heterozygous. Twenty-nine and 11 variants involved IEI-related genes associated with recessive and dominant traits, respectively; the remaining 3 affected recessive genes have also been previously linked to atypical IEI in heterozygous status (Figure 2, C and D). Gene-phenotypic analysis clustered the variants in combined T/B cell defects (*n* = 12), immune dysregulation/autoinflammation (*n* = 10), bone marrow failure (BMF, *n* = 6), phagocyte defects (*n* = 6), B cell defects (*n* = 5), and innate immunodeficiency (*n* = 4) (Figure 2E). The gene variants linked to immune dysregulation and innate or humoral immunodeficiency syndromes were more commonly associated with dominant and adult-onset IEI traits (Figure 2F) (19, 22).

### Characterization of T-LGLL patients with IEI high-confidence deleterious variants.

**Figure 3:**
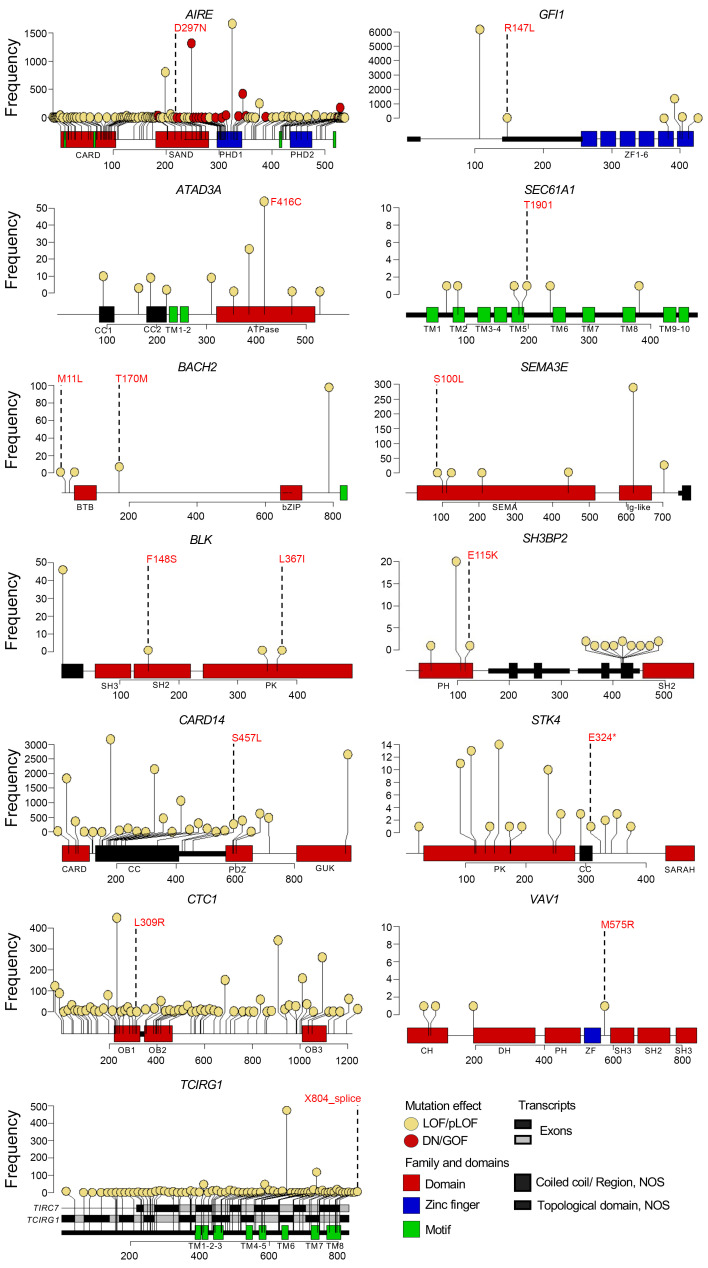
Mutations in 13 IEI genes with hcD variants identified in T-LGLL patients. Annotation of the domains of the proteins coded by the canonical transcripts was extracted from Ensembl and UniProt.json files. For TCIRG1, exon-protein correlations for both the canonical and alternative transcripts are shown. The mutations labeled in red with the amino acid change are the ones found in our study. The plot additionally displays rare (MAF < 1%) deleterious variants previously reported in these genes using gnomAD genomic browser, version 4.1.0, integrating pathogenicity predictors and variant frequency (number of variants reported in gnomAD). LOF/pLOF, loss of function/predicted LOF; NOS, region/domain not otherwise specified.

To further establish biological-clinical correlations, we defined carriers of high-confidence deleterious (hcD) variants, more likely to predispose to immune misbalance, as those harboring either P/LP defects, or heterozygous VUS for dominant traits. Fifteen (16%) of 92 patients carried these high-risk variants involving 13 genes (Figure 3, Supplemental Figures 1–3, and Table 2). Among them, the only case with a genetic diagnosis of an IEI was included: a 61-year-old man with a history of autoimmune hemolytic anemia prior to T-LGLL who had a heterozygous P/LP variant in *AIRE* (p.R297N). Biallelic mutations in this master regulator of central tolerance are associated with autoimmune polyendocrine syndrome type 1 (APS-1, OMIM#607358). However, heterozygous mutations involving the *plant homeodomain* (PHD) region, where R297 is located, can lead to atypical APS-1, characterized by late-onset, organ-specific autoimmunity due to a dominant-negative effect of the altered allele (23). A case of biallelic, classic APS-1 with T-LGLL was previously reported in a 34-year-old woman with concurrent pure red cell aplasia (PRCA) (24).

We also found a heterozygous P/LP variant in *TCIRG1* (c.1767-2A>G) in a 66-year-old man with chronic neutropenia and personal and family history (mother) of rheumatoid arthritis (Figure 3, Supplemental Figures 1–3, and Table 2). Biallelic mutations involving this phagocytic H^+^-ATPase cause recessive malignant osteopetrosis (OMIM#604592), while heterozygous variants have been associated with dominant congenital neutropenia. This splice site variant, involving the 3′ (acceptor) AG sequence of *TCIRG1* intron 14, is predicted to have a significant, negative effect by different algorithms, including the MaxEntScan (25) model for splice site variants (score 1.91, mutant-WT Δ=7.95 –predicted as a high potential to disrupt native splicing). Notably, the same gene, through usage of an alternative initiation codon in exon 7, generates TIRC7, a different protein expressed by T cells with immune checkpoint functions (26). The location of this variant is predicted to impair the splicing of the 2 proteins, thus potentially explaining both the neutropenia and the autoimmune manifestations in this case.

The patient with the earliest diagnosis of T-LGLL within our cohort was also found to carry a hcD defect: a 17-year-old man with a prior history of CVID, who had a complicated debut mimicking severe immune-mediated BMF, and undergoing allogeneic hematopoietic stem cell transplant (Figure 3, Supplemental Figures 1–3, and Table 2). He carried a heterozygous P/LP variant in *STK4* (p.E324*), linked to combined T/B cell defects, lymphoproliferation, and cytopenias in biallelic configuration (OMIM# 614868). The patient was also a heterozygous carrier of a hypomorphic, low-frequency variant in *PRF1* (p.A91V, MAF = 0.0293), which has been described as a risk factor for late-onset hemophagocytic lymphohistiocytosis, acquired aplastic anemia, and different T cell lymphoid neoplasms (27, 28). Peripheral blood flow cytometry performed at the referring institution before transplant revealed decreased proportions of NK and NK/T cells expressing perforin as described for hemophagocytic lymphohistiocytosis (51% [normal:86%–98%] and 2% [normal:30%–78%], respectively), suggesting combinatory effects of the variants identified in this case.

Among the other cases with hcD variants, we point out 2 patients with heterozygous gene defects in *BACH2* (Figure 3, Supplemental Figures 1–3, and Table 2). A dominant, adult-onset IEI has been recently linked to this gene, characterized by lymphocyte maturation defects, intestinal inflammation, and hypogammaglobulinemia (29). Both cases from our cohort were diagnosed early with T-LGLL, in the fourth decade of life; they had personal and family history of autoimmune disease and presented CD4^+^ T cell defects. One of them exhibited all the features described for BACH2 deficiency syndrome: a 36-year-woman with a history of type 1 diabetes, inflammatory bowel disease, PRCA, and IgA deficiency prior to T-LGLL diagnosis. This patient was found to carry a heterozygous missense variant involving the dimerization domain (p.M11L), close to previously described P/LP variants (29).

### Distinct clinical features identified in carriers of IEI hcD variants.

**Figure 4:**
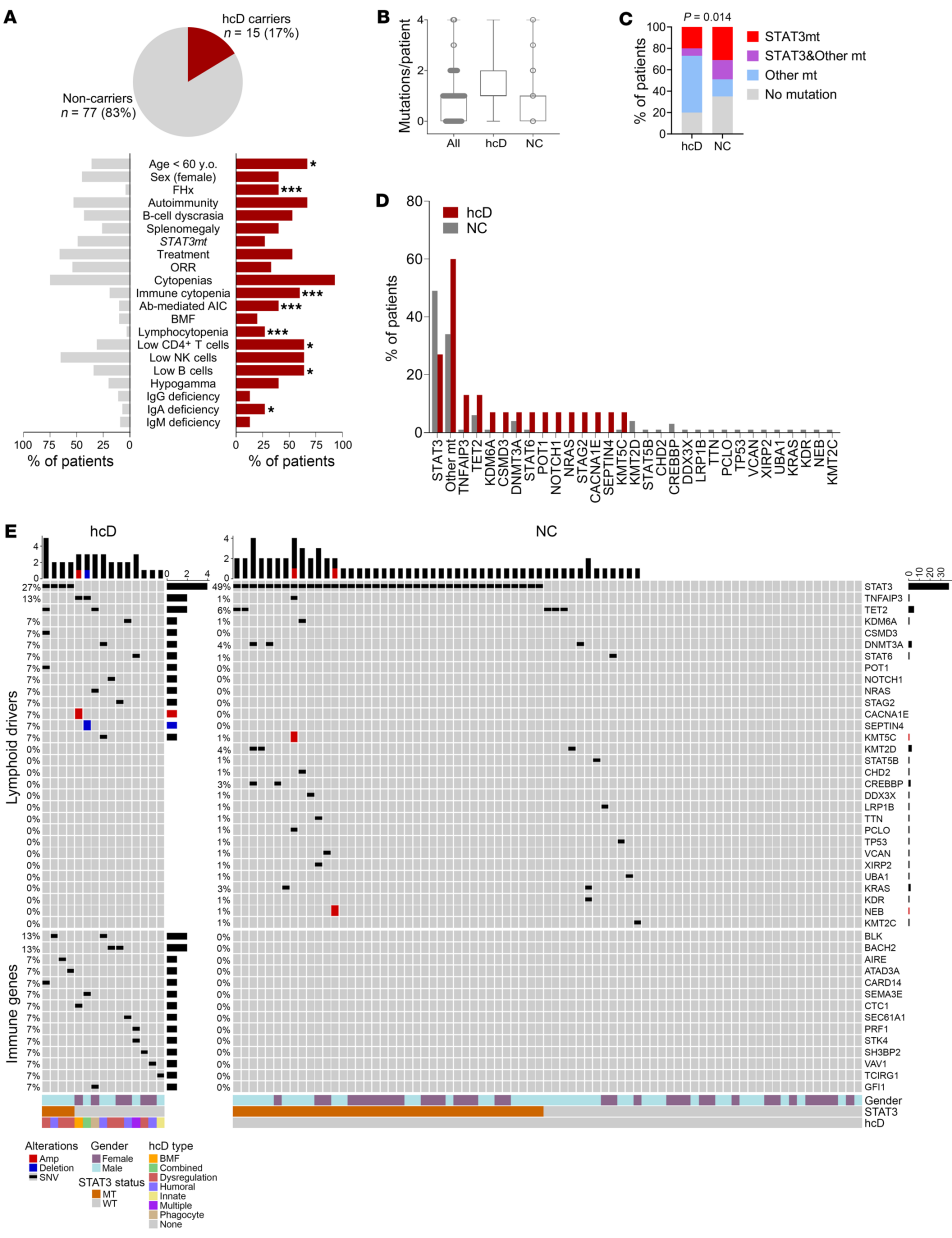
Clinical, laboratory, and genetic characterization of patients with hcD IEI variants. (**A**) Clinical and laboratory features of the carriers of hcD variants (hcD, red) versus noncarriers (NC, gray). Family history (FHx) designates positive family history for immune or hemato-lymphoid conditions. The category immune cytopenia includes both BMF (i.e., aplastic anemia or PRCA) and antibody-mediated peripheral autoimmune cytopenias (Ab-mediated autoimmune cytopenia, i.e., autoimmune hemolytic anemia, autoimmune neutropenia, or immune thrombocytopenia). (**B**) Mutational burden (mutations/patient). (**C**) Mutational configuration in *STAT3* and other T cell lymphoid driver genes. (**D**) Frequency (%) of mutations in individual T cell lymphoid driver genes. (**E**) Mutational profile in carriers and noncarriers of hcD variants. The upper plot represents somatic mutations in T cell lymphoid driver genes. The lower plot shows the germline hcD variants in IEI-linked genes, clustered by immune pathways. The specific mutated IEI gene is detailed for each case. The low-frequency, hypomorphic PRF1 variant identified in P49 is also shown. Main clinical features and color legends are indicated below. χ^2^ *P* values are shown. *P < 0.05; **P < 0.01; ***P < 0.001. Ab-mediated AIC, antibody-mediated peripheral autoimmune cytopenia; ANC, absolute neutrophil count; FHx, family history of immune/hemato-lymphoid conditions; ORR, overall response rate; y.o., years old.

Next, we investigated the clinical and laboratory features of carriers of hcD variants (Figure 4A and Supplemental Figure 4). As compared with noncarriers, these patients were diagnosed with T-LGLL earlier (median: 55 versus 64 years, *P* = 0.01) and more frequently had a family history of immune or hemato-lymphoid conditions (40% versus 4%, *P* < 0.001). Lower lymphocyte and large granular lymphocyte (LGL) counts were appreciated, resulting in higher rates of lymphocytopenia (27% versus 3%, *P* = 0.006). Carriers had notably higher frequencies of hypogammaglobulinemia (40% versus 20%) and cytopenias of any type (93% versus 75%), although the significance level was not reached, probably due to relatively small sample size (statistical power = 60%). However, they had significantly higher rates of IgA deficiency (27% versus 7%, *P* = 0.03) and autoimmune cytopenias or BMF (60% versus 19%, *P* = 0.001) than noncarriers.

There were no differences in the need of LGLL therapy, nor in the overall response rate as severity indicators. In addition, survival curves suggested an apparent favorable trajectory for hcD carriers that, however, was not confirmed in multivariate analysis (Supplemental Figure 5 and Supplemental Table 3).

### Mutational landscape in T-cell lymphoid drivers.

Analysis of single-nucleotide variants (SNVs) and copy-number variants (CNVs) in T cell lymphoid drivers (169 genes, Supplemental Table 4) revealed 91 variants of clinical relevance in 30 different genes in 62 (67%) of 92 patients (Supplemental Table 5). Mean mutational burden was 1 (range: 0 – 4 mutations/patient, Figure 4B). Mutations in genes other than *STAT3* were detected in 45 (49%) of 92 patients. Mutations in *STAT5B* and *STAT6* were identified in 3 (3%) patients. Mutations in epigenetic modifiers *KMT2C*, *KMT2D*, *KDM6A*, *KMT5C*, *TET2*, *DNMT3A* and *CREBBP* were detected in 16 (17%) patients, in 9 of 16 (56%) of them in cooccurrence with *STAT3* mutations. We also identified variants in other recurrent mature T cell neoplasm genes, such as the RNA helicase *DDX3X* and the tumor suppressor *TP53* — one case each. Other oncogenes/tumor suppressors harboring damaging mutations in our cohort were *CSMD3*, *POT1*, *NOTCH1*, *NRAS*, *STAG2*, *CACNA1E*, *SEPTIN4*, *CHD2*, *LRP1B*, *TTN*, *PCLO*, *VCAN*, *XIRP2*, *UBA1*, *KRAS*, and *KDR*. As expected for our focus on T-LGLL, we did not find mutations in NK-cell lymphoid neoplasm genes *CCL2* or *CCL22* (Figure 4, C–E and Supplemental Table 5).

Gene-level CNV analysis conducted in WES data did not detect the presence of copy number (CN) gains in *STAT3*, *STAT5A*, or *STAT5B* (located in chromosome [chr] 17q21.2), nor CN losses in *TP53* (chr 17p13.1, Supplemental Figure 6). We first focused on the screening for CNVs in these genes because of their being common CNV hotspots in other T cell neoplasms (30–33), as confirmed by the interrogation of genomic data from 26 human T cell cancer cell lines gathered from DepMap (Supplemental Table 6 and Supplemental Figures 7 and 8) (34). In contrast with T-LGLL, assessment of SNVs and CNVs in T cell cancer cell lines showed alterations in *STAT3* via CN gain in 3 (12%) of 26 cell lines and *TP53* SNV and CN loss in 15 (58%) and 5 (19%) of 26 cell lines, respectively. Next, we extended our analysis of CNVs to other T cell lymphoid drivers, which yielded alterations in 4 (4%) of 92 T-LGLL patients (Supplemental Table 7), including the amplification of *CACNA1E* (chr 1q25), *NEB* (chr 2q23) and *KMT5C* (chr 19q13), and the loss of *SEPTIN4* (chr 17q22, this latter case not including the *STAT3* locus).

Comparison of IEI hcD carriers versus noncarriers revealed significantly different mutational configurations (*P* = 0.014): 4 of 15 (27%) versus 38 of 77 (49%) cases were *STAT3*mt; conversely 9 of 15 (60%) versus 26 of 77 (34%) had mutations in genes other than *STAT3*; 1 of 15 (7%) versus 14 of 77 (18%) patients had coexistence of *STAT3* and other gene mutations. Among the genes other than *STAT3* mutated in *STAT3*wt hcD carriers, we noted hotspot mutations in *TNFAIP3* (*n* = 2), *NOTCH1* (*n* = 1), and *NRAS* (*n* = 1) as well as CNVs in *CACNA1E* and *SEPTIN4* in the 2 patients with mutations in *TNFAIP3* (Figure 4, C–E).

### Comutation analysis of IEI and T cell lymphoid gene variants.

Next, we sought to explore genetic-biological correlations. Gene ontology (GO) term pathway analysis revealed that the hub formed by the IEI-related genes having hcD variants had 28 enriched commonalities with the network formed by a list of known T-LGLL dysregulated genes (Supplemental Figure 9A and Supplemental Table 8) (35). Among them, cellular response to biotic stimulus, pathogen recognition, production/regulation of IL-12 and TNF-α, cellular response to IL-6, or positive regulation of T cells was encountered, supporting the pathogenic role of hcD variants in promoting CTL expansion via aberrant/defective signals involving these pathways.

The IEI hcD variants can be phenotypically dichotomized according to the type of the abnormal response as immune dysregulation (hyperactive response) or innate/adaptive immunodeficiency (defective response) defects. To further investigate the interplay between IEI hcD and T cell driver genes, we performed variant correlation and HALLMARK (https://www.gsea-msigdb.org/) pathway enrichment analysis. We observed an enrichment of mutations in elements of the IL-6/JAK/STAT3, NOTCH, and TP53 pathways in cases with IEI variants associated with immune dysregulation. In contrast, there was enrichment of mutations associated with autoimmunity and lymphoproliferation via TNF-α/NF-κB and the RAS pathways in cases with IEI variants associated with immunodeficiency (Supplemental Figure 9, B and C).

### RNA-Seq analysis of TCR signaling pathway in STAT3mt versus STAT3wt expanded T cells.

Chronic antigen stimulation has been suggested to initially drive the outgrowth of T-LGL clones. However, there are limited data supporting the role of T cell receptor (TCR) signaling in the disease. Based on a lower *STAT3*mt rate in IEI hcD carriers, we hypothesized that the clonal expansion might rely on TCR signaling more strongly in *STAT3*wt versus mutant cells, as described for other T cell lymphoid neoplasms having *STAT3*mt (36–39). To this end, 2 transcriptomic datasets were interrogated: (a) single-cell RNA+TCRαβ-Seq (scRNA-Seq + TCRαβ-Seq) of T-LGLL (*n* = 11) and healthy control (*n* = 6) samples, independently repurposed from the study by Huuhtanen and Bhattacharya et al. (Supplemental Table 9) (13) and (b) bulk RNA-Seq of mature T cell cancer cell lines from DepMap (*n* = 22, Supplemental Table 6, Supplemental Figure 8) (34).

Gene-expression levels and differential expression analyses were performed in *STAT3*mt versus *STAT3*wt hyperexpanded T cells (>10 TCR templates) from scRNA-Seq+ TCRαβ-Seq samples (Supplemental Figure 10). We observed that several genes proximal to the TCR complex were differentially expressed between *STAT3*mt and *STAT3*wt clones. Specifically, *TRBC* and *TRBV* genes, *CD2*, *CD3E*, *CD5*, *CD8A*, *LAT*, *LCK*, *SYK*, or *ZAP70* were upregulated in *STAT3*wt cells (i.e. downregulated in *STAT3*mt clones), which showed expression levels like those of hyperexpanded T cells from healthy controls (Supplemental Table 10 and Supplemental Figures 11 and 12). Previous differential gene-pathway analysis of this experimental dataset also suggested upregulation of the TCR signaling pathway in *STAT3*wt versus *STAT3*mt cells (13). Next, to validate these findings, we analyzed the expression of TCR-related genes in 22 T cell cancer cell lines according to the *STAT3* amplification status previously assessed (*STAT3*-amplified [*STAT3*amp] *n* = 3; *STAT3*wt *n* = 19, Supplemental Figures 7 and 8). We confirmed that *STAT3*wt versus *STAT3*amp cells had generally lower expression of TCR-related genes, including *CD3Z/CD247*, *ZAP70*, *LCK*, and *LAT* (Supplemental Figure 13). Differential expression and pathway analysis (40) of *STAT3*amp versus fusion-matched *STAT3*wt lines (Supplemental Methods and Supplemental Tables 6 and 11) also showed significant upregulation of immune receptor–based signaling pathways in *STAT3*wt versus *STAT3*amp cells (Supplemental Figure 14).

**Figure 5:**
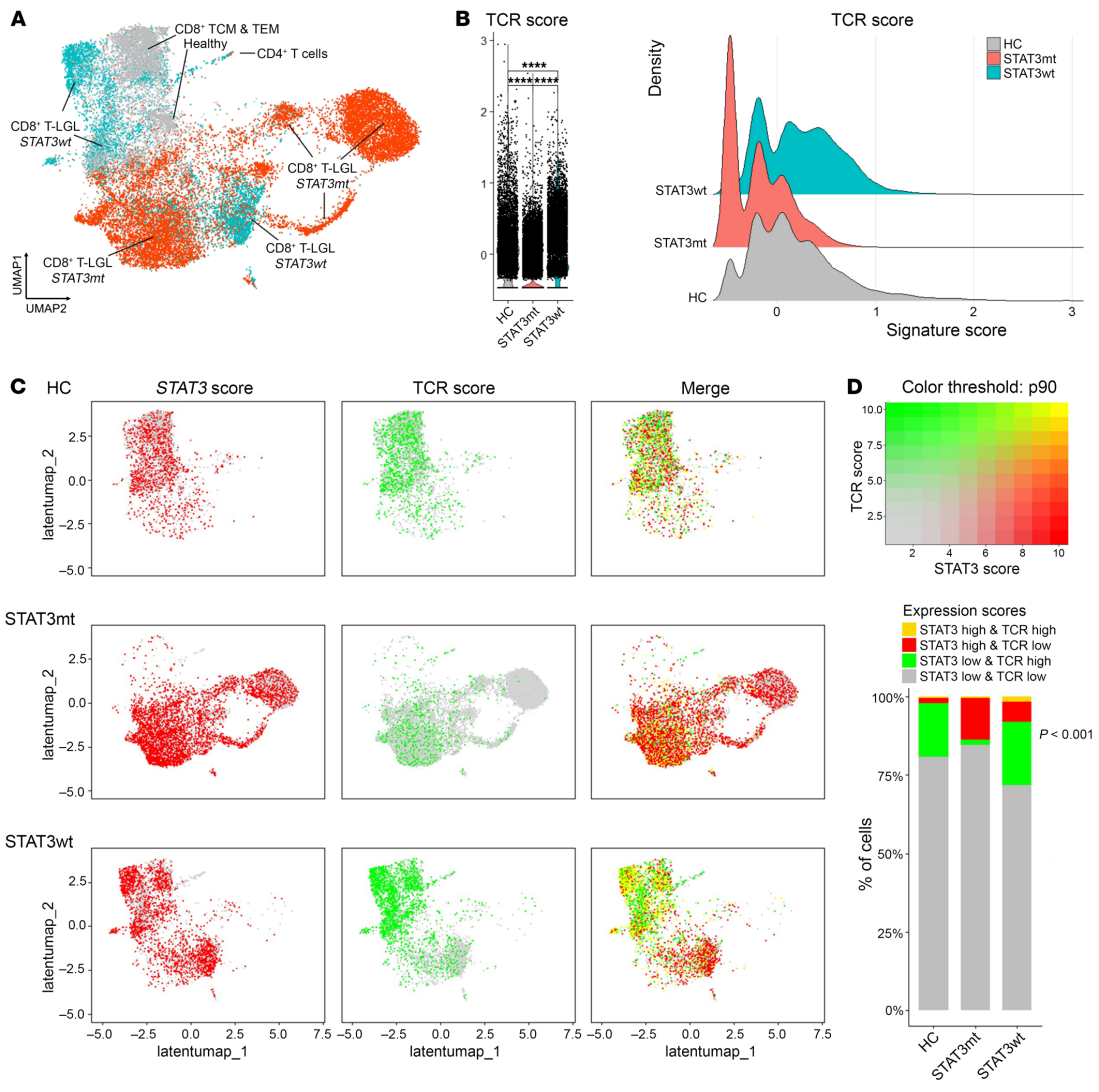
Single-cell RNA+TCRαβ expression analysis of TCR signaling genes in *STAT3*mt and *STAT3*wt cells from T-LGLL samples and healthy controls. (**A**) UMAP representation of the reclustered hyperexpanded T cells (>10 templates) annotated by cell phenotype and STAT3mt status. (**B**) TCR signalosome score in *STAT3*mt versus *STAT3*wt cells. The violin and ridge plots show the TCR score in *STAT3*mt and *STAT3*wt T-LGLL clones and in healthy control hyperexpanded T cells. Wilcoxon’s test *P* < 0.10 are shown. (**C**) Coexpression analysis of TCR and *STAT3* signaling scores split by the type of cell. STAT3mt status and scaled expression of *STAT3* activation (red) and TCR signalosome scores (green) are highlighted in the same UMAP representation. Color scale corresponds to the signature score, and the color thresholds correspond to the 90th percentile of the scores. The cells are divided as *STAT3*mt, *STAT3*wt, and healthy controls. Gray dots correspond to *STAT3* and TCR expression below the 90th percentile; red and green signals correspond to strong *STAT3* and TCR signaling, respectively; yellow signals correspond to double STAT3/TCR-high signaling. (**D**) Proportion of cells with high values of *STAT3* and TCR signaling scores according to the STAT3mt status. χ^2^ *P* values are shown. ****P < 0.0001. HC, healthy controls.

To further investigate the interplay between *STAT3*mt status and TCR signaling, we calculated a TCR signaling expression score using the Seurat AddModuleScore function (41). To this end, we used 15 TCR-related genes, including *TRAC* and *TRDC* as constant elements of the TCR complex (GO:0042101) and components of the TCR signalosome (GO:0036398). A score with the same set of genes was also calculated in bulk RNA-Seq from the T cell cancer cell lines as geometric means as previously described (13). In T-LGLL samples, *STAT3*mt clones had significantly lower TCR scores than *STAT3*wt and healthy control cells. Similarly, lower TCR scores were found in *STAT3*amp versus *STAT3*wt T cell cancer cell lines (Figure 5, A and B, and Supplemental Figure 15).

T-LGLs correspond to mature T cells with generally high expression of TCR. Therefore, to assess the relationship between strong TCR and STAT3 signaling in a single-cell basis, we categorized the TCR score as high (above the 90th percentile) or low (below the 90th percentile) and compared the distribution of these TCR-high/low cells depending on the *STAT3*mt status. In addition, using the lists of genes upregulated in *STAT3*mt versus *STAT3*wt cells generated here and gathered from MSigDB (42), we calculated a 9-gene STAT3 signaling score as an indicator of STAT3 activation (Supplemental Methods). Grouping of the cells as STAT3 score high/low was also done using the 90th percentile threshold. Coexpression analysis showed that STAT3-high scores were overrepresented in the *STAT3*mt clones (14% versus 8% and 2% in *STAT3*wt and healthy control cells, respectively, *P* < 0.0001, Figure 5, C and D), while TCR-high scores were more frequent in *STAT3*wt and healthy cells (22% and 17% versus 2% in *STAT3*mt clones, respectively, *P* < 0.0001). Overall, cells with double STAT3-high/TCR-high scores were infrequent (0.71%). Altogether, our results suggest that the TCR signaling might have a pathogenic contribution on CTL outgrowth, particularly prior to the acquisition of GOF *STAT3* mutations during T-LGLL evolution.

### TCR repertoires and target specificities in T-LGLL samples.

**Figure 6:**
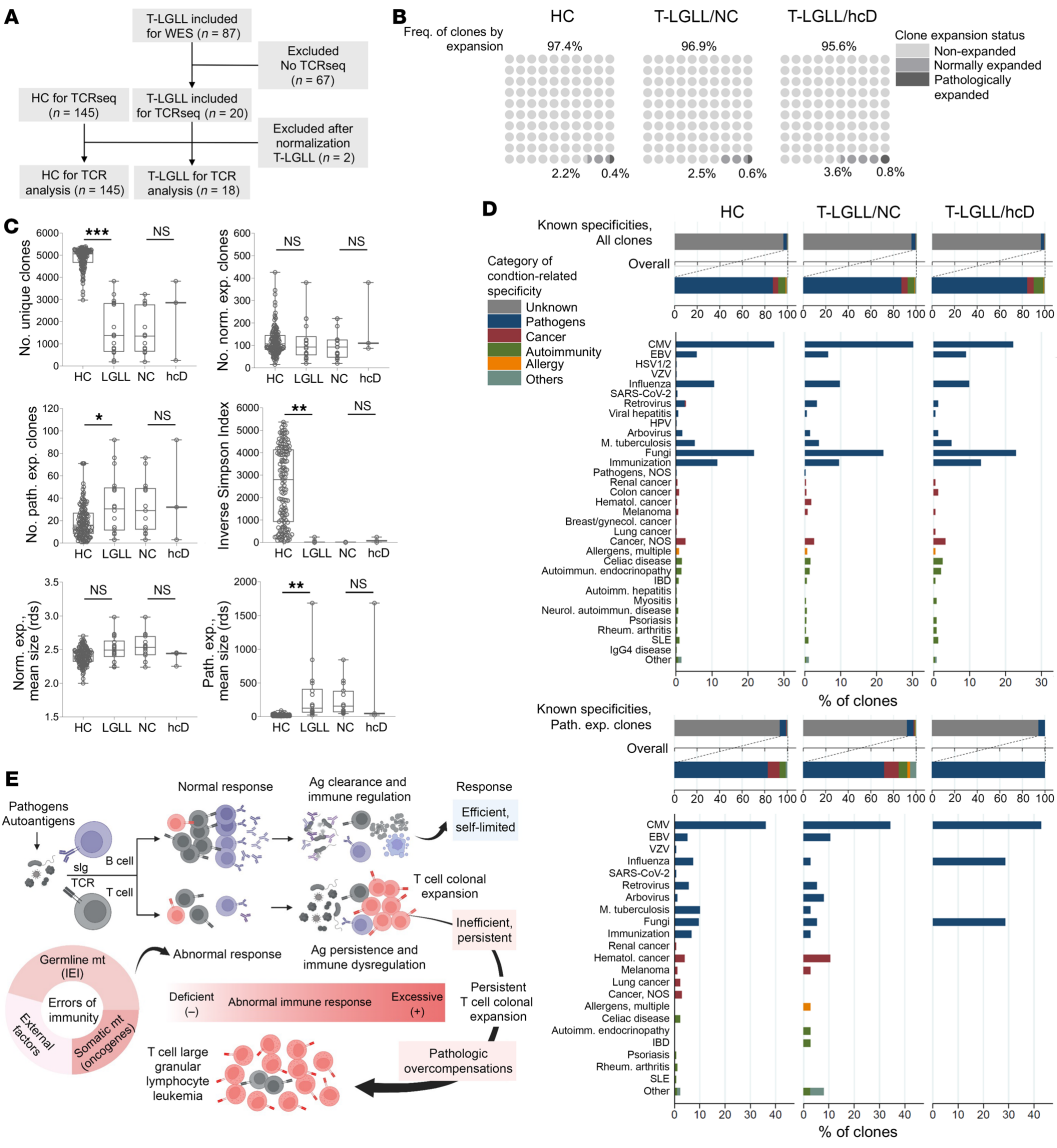
TCR repertoire analyses of the implications of the hcD variants associated with dominant IEI. (**A**) Patient flow for TCR immunosequencing. Of 20 T-LGLL patients with WES and deep TCR sequencing, 18 patients were downstream analyzed after resampling to 5,420 clones. Data from 145 healthy controls from Pagliuca et al. were also used (45). (**B**) Pooled distribution of clones according to the expansion status. Nonexpanded, normally expanded, and pathologically expanded clonotypes are defined by 1, 2–5, and >5 templates, respectively. (**C**) Number of unique, normally expanded, and pathologically expanded clonotypes, inverse Simpson index, and mean clone size between T-LGLL versus healthy controls and between hcD carriers and noncarriers (NC) within the T-LGLL cases. One dot per sample. Mann-Whitney *U* test *P* values are shown. (**D**) Bar plot illustrating the condition-related known specificities of the whole repertoire (upper plot) and only the pathologically expanded clonotypes (lower plot) in healthy controls and T-LGLL patients with/without hcD variants. (**E**) Proposed model on the pathogenic role of underlying IEI in CTL proliferations and clonal shift (created with Biorender.com). Aberrant immune responses caused by genetic or acquired factors, both deficient or hyperreactive abnormal responses, may lead to antigen persistence and/or immune dysregulation and eventually result in T-LGLL as a pathologic overcompensation. *P < 0.05; **P < 0.01; ***P < 0.001. Ag: antigen; CMV, cytomegalovirus; IBD, inflammatory bowel disease; HPV, human papillomavirus; NOS, not otherwise specified; sIg, surface Ig; SLE, systemic lupus erythematosus; VZV, varicella zoster virus.

Next, to further investigate the diversity and the nature of the T cell responses promoted, we studied the TCR repertoire using deep variable β (VB) complementarity determining regions (CDR3) sequencing. We analyzed productive rearrangements within normalized repertoires in a subset of 18 T-LGLL patients, 3 of whom carried hcD variants associated with innate/adaptive immunodeficiency (Figure 6A). As compared with the repertoires of 145 healthy controls, normalized in a similar fashion (43, 44), all metrics supported a lesser level of TCR diversity. Consistent with the diagnosis, we also found a higher number and size of pathologically expanded clonotypes in T-LGLL patients. However, no substantial differences in these parameters were appreciated between carriers and noncarriers of hcD variants (Figure 6, B and C). In an effort to track the possible targets of immune responses, we then assessed clonotype specificities according to our previously published metanalytic collection of CDR3b sequences (Supplemental Table 12) (45). Initial analysis of the whole repertoires revealed exact matches with the reference in 4% of the sample clonotypes, with similar proportions between groups associated with response to infection, autoimmunity, and tumor surveillance. Analysis focused on pathologically expanded clonotypes showed exact matches in 6% clones; most of these specificities (185/227, 81%), and all found in T-LGLL patients with hcD variants (7/7, 100%), were targeted against pathogens, supporting the notion that microbial agents might be the prevailing antigenic triggers in these cases (Figure 6D).

A graphical summary of the proposed model on the pathogenic role of underlying IEI in CTL proliferations and clonal shift is shown in Figure 6E.

## Discussion

Several recent advances have been made toward a better understanding of the pathogenesis of T-LGLL (7), for instance, the identification of mutations in genes other than *STAT3* (e.g., *STAT5B*, *TNFAIP3*, epigenetic regulators) or the role of the immune microenvironment besides the leukemic clone (13). Chronic antigen stimulation has been classically hypothesized to initiate/drive CTL outgrowth, based on both clinical and translational observations (8). There is, however, limited direct evidence on the role of antigen recognition in T-LGLL or on the identity of the culprit(s) epitope(s). Indeed, no common TCR sequences have been identified across T-LGL clones of different patients (13). However, this does not contradict that T-LGLL can be driven by an abnormal response to an antigen. For instance, it has been shown that, in a significant fraction of T-LGLL patients, the leukemic clones have been found to share CDR3 motifs with their nonleukemic counterpart (13, 46). An increased costimulatory cell-cell and cytokine crosstalk with nonleukemic lymphocytes and antigen-presenting cells has also been reported (13). Altogether, current evidence suggests that, despite the fact that the triggering event might be caused by different epitopes, common mechanisms independent of the inciting antigen may be at the root of the disorder.

In this line, we systematically explored in one of the largest genomic T-LGLL cohorts the possibility that the initial overshooting immune response in T-LGLL might be specifically induced in individuals genetically predisposed to errors of immunity. Overall, our results suggest that IEI variants are found in a substantial fraction of T-LGLL cases. Prior to this work, 6 cases of T-LGLL in severe IEI were reported, including 4 cases with recessive SCID (18, 47–49), a patient with X-linked agammaglobulinemia (50), and a case with APS-1 (24). Among them, we highlight the report of 2 identical twins affected by ADA2 deficiency who developed *STAT3*wt T-LGLL as a consistent manifestation with a staggered time course (49). Importantly, T-LGLL is a typical disease of the elderly, making the diagnosis of such severe syndromes during adulthood very unlikely. Conversely, the defects we found here were all heterozygous and mostly associated with dominant, adult-onset IEI, supporting their pathogenic role (22). As compared with classic IEI, and as expected for the growing family of adult-onset IEI, less-deleterious variants for dominant traits and monoallelic variants for recessive diseases were found in our study, probably explaining incomplete penetrance, variable expressivity, and delayed or atypical manifestations.

Despite IEI constituting a growing and highly heterogeneous family of disease, these syndromes can be phenotypically dichotomized as per an excessive or defective immune response (i.e., immune dysregulation and innate/adaptive immunodeficiency syndromes, respectively) (19, 22). Interestingly, both IEI categories were represented by the hcD variants found in the T-LGLL WES cohort, suggesting 2 routes by which IEI may promote CTL expansions: (a) immune dysregulation, intrinsically overshooting autoimmunity, and (b) innate/adaptive immune defects, probably due to increased risk of infection or inability to efficiently clear pathogens. Therefore, both deficient and excessive abnormal immune responses might fuel inefficient, persistent CTL outgrowth. A baseline compromised T cell repertoire may additionally predispose to the oligoclonal/clonal shift. In this study, we found that carriers of IEI hcD variants might exhibit distinct features, such as early T-LGLL diagnosis, low lymphocyte counts, low rates of somatic *STAT3* mutations, and high proportions of hypogammaglobulinemia, autoimmune cytopenias, or BMF. These atypical features reinforce an alternative pathogenesis of modifying genetic factors present in a fraction of cases with T-LGLL. Importantly, in the clinical setting, they may serve as red flags tied to the underlying/cryptic IEI. A better characterization of the immune defects in a patient may open the possibility of using supportive measures (e.g., antimicrobial prophylaxis, Ig replacement) or tailored therapies as well as avoiding multiple lines of ineffective treatment. Ultimately, the accurate diagnosis of a cryptic IEI in young adults with refractory severe cytopenias may lead to evaluation of hematopoietic stem cell transplant as a definitive treatment. Multidisciplinary care is recommended in this complex patient population (51).

In this study, we comprehensively characterized the mutational landscape of T-LGLL. We identified SNVs and CNVs in T cell lymphoid drivers in 67% patients. Mutations in genes other than *STAT3* were detected in 49% of patients, including validated targets such as *STAT5B*, *TNFAIP3*, and epigenetic regulators. We also identified SNVs and CNVs in recurrently mutated genes in T cell neoplasms and lymphoid clonal hemopoiesis. Amplification of *STAT3* and *STAT5A/B* and deletion of *TP53* are frequent events in T cell leukemia/lymphomas, particularly after relapse/transformation as well as in cell lines due to increased genomic instability (30–32, 52). We have confirmed this in a comprehensive analysis of 26 T cell cancer cell lines from DepMap. In contrast, analysis of our WES cohort did not identify such CNVs in T-LGLL samples. We identified, however, CNVs in other T cell lymphoid drivers in 4% of cases. This is consistent with a recent study on 105 T-LGLL patients using WES, which evidenced a similar rate of CNVs (5). The use of whole-genome sequencing, third-generation sequencing based in long-reads, or optical genome mapping would be necessary in future studies to better assess the full CNV spectrum of T-LGLL (53, 54).

Comparison of IEI hcD carriers versus noncarriers revealed different mutational configurations. We highlight a lower proportion of *STAT3*mt cases in carriers than noncarriers. Based on this finding, we investigated the interplay between TCR signaling and *STAT3*mt status by using scRNA+TCR-Seq of T-LGLL samples and RNA-Seq of T cell cancer cell lines. Our genomic T-LGLL cohort did not have coupled RNA-Seq for integrated expression analysis. However, both RNA-Seq datasets utilized instead here allowed the possibility to accurately separate and compare *STAT3*mt versus *STAT3*wt cells. Gene-expression levels, differential expression, and pathway analyses consistently suggested downregulation of TCR-related genes in *STAT3*mt T-LGLs, which were upregulated in *STAT3*wt clones. An analogous bypass of TCR complex signaling by enhanced STAT3 activation has been described in T cell lymphoid neoplasms of other subtypes and was confirmed in our analysis of RNA-Seq of T cell cancer cell lines (36–39). This finding provides more indirect evidence supporting the role of TCR signaling in T-LGLL pathogenesis. Notably, *STAT3*mt is considered a subclonal, late event occurring in the context of an initial oligoclonal/clonal T cell expansion (6, 7, 55). Therefore, TCR signaling might not only be important in *STAT3*wt patients, but also in *STAT3*mt cases prior to the acquisition of the GOF mutation and even after that to support the continuous growth of pathologically expanded *STAT3*wt clones.

Apart from a lower *STAT3*mt rate, IEI hcD carriers had a greater proportion of mutations in genes other than *STAT3* (60%), including an enrichment in hotspot mutations in *TNFAIP3*, *NOTCH1*, and the RAS pathway. Somatic mutations in these genes have been associated with acquired immune dysregulation and lymphoproliferation syndromes. Similar results have been published in a cohort of 17 patients with primary immunodeficiency interrogating somatic mutations in T cells (18, 56). Illustratively, in this study, one patient with SCID due to ADA2 deficiency and *STAT3*wt T-LGL expansion was found to carry a somatic mutation in *KRAS*. Altogether, these findings point toward different molecular configurations driving the T cell expansion resulting in T-LGLL. For patients with variants predisposing to errors of immunity, this might constitute an example of a maladaptive somatic gene rescue, a phenomenon described for patients with SCID or with inherited BMF syndromes (57–60).

Our study has several limitations. The assessment of the functional impact of the numerous variants identified through genomics is an important step in searching for disease-causing mutations. Given the heterogeneity and diverse mechanism across IEI, we approached this issue by (a) selecting variants overrepresented in our cohort (significant *P* value after multiple-testing correction); (b) using the ACMG criteria for unified analysis; (c) testing the variants by using different in silico prediction tools; and (d) stratifying the analysis of the variants based on pathogenicity, validated phenotypic classifications, and disease inheritance and/or age of onset. Establishment of gene-phenotype correlations can also be challenging, in particular when applied to rare diseases. Therefore, we screened a panel of immune genes only included in the IUIS consensus of IEI (19), an updated classification whereby genotypic data are accompanied with clinical and laboratory phenotypic features. However, given that the selection of genes is empiric, we acknowledge that the list may not be complete. In addition, and most importantly, acquired immunodeficiency due to comorbidities or age-related decline in immune function may also be causative. Assessment of the TCR clonotyping was also limited by the relatively low sample size and the lack of in vitro validations. Specificities were assessed both in the whole repertoire and pathologically expanded clonotypes. Despite the relatively high number of clonotypes included in the reference (>80,000), the proportion of exact sample-reference matches was low (≈5%), a finding that is, however, expected for this approach. Among the matches, most of them were associated with infectious agents, indirectly supporting the notion that microbes might be the prevailing antigenic drivers in some of these cases.

Our study was focused on T-LGLL. Cases with NK cell proliferations were excluded because, although unified by some features, such as *STAT3* mutations, NK-LGLL constitutes a less frequent (<10% of LGLL cases), and less clinically/molecularly defined variant of the disease (7). The diagnostic boundaries between clonal and nonclonal NK cell expansions are blurred, and mutations in *CCL22*, characteristic of NK-LGLL (52, 61), have not been reported in T-LGLL cases, as confirmed by our genomic assessment. Future studies conducted in multicenter, collaborative cohorts should be designed to characterize the immunogenetic landscape of NK-LGLL.

In conclusion, our study proposes an alternative pathogenesis for T-LGLL paradoxically related to underlying seemingly indolent errors of immunity. These, under certain circumstances, might lead to aberrant CTL responses. Altogether, our findings constitute an illustrative lesson of nature on the understanding of clonal CTL expansions and open the horizon of IEI to other clonal hematological disorders and immune-mediated BMF.

## Methods

Full description of methods is available in the Supplemental Methods.

### Sex as a biological variable

This study examined male and female participants, as both men and women were eligible, and findings were similar for both sexes.

### Clinical cohort

This cross-sectional, genetic association study was performed in a cohort of consecutive patients with T-LGLL, diagnosed and managed at Taussig Cancer Center, Cleveland Clinic Foundation from 1998 to 2023, described elsewhere (Table 1) (2). Because of our focus on T cell clonal expansions, cases with NK-LGLL subtype were excluded. Clinical data collected comprised patient demographics, comorbidities, personal and family history of immune disorders or hemato-lymphoid neoplasms, splenomegaly, cytopenias, and BMF (62). Conditions potentially leading to hypogammaglobulinemia (63) were screened in patient’s medical charts prior to T-LGLL diagnosis. Clinical outcomes analyzed were treatment initiation, transfusion dependency, splenectomy, hematopoietic stem cell transplant, transformation to high-grade lymphoma, or death.

Laboratory data at diagnosis comprised complete blood count, LGL count, Ig levels, and M protein assessment. Lymphocyte subset characterization by flow cytometry (in CD45^Ly^ gated cells) routinely performed in the clinic in peripheral blood at LGL diagnosis was also collected for the study of the following populations: T cells (CD3^+^, normal range [NR]: 0.96–2.39 × 10^9^/L), CD4^+^ T helper cells (CD3^+^CD4^+^, NR: 0.53–1.67 × 10^9^/L), CD8^+^ cytotoxic T lymphocytes (CTLs, CD3^+^CD8^+^, NR: 0.28–0.96 × 10^9^/L), NK cells (CD3^–^CD16/CD56^+^, NR: 0.10–0.57 × 10^9^/L), and B cells (CD19^+^, NR: 0.08–0.66 × 10^9^/L). The Ig levels analyzed included the quantification of IgG (NR: 717–1411 mg/dL), IgA (NR: 78–391 mg/dL), and IgM (NR: 53–334 mg/dL).

Targeted sequencing was performed as previously described using a custom panel for detection of hematological neoplasm gene variants from TruSeq or Nextera platforms (Illumina) (64, 65). *STAT3* mutational status was assessed by deep DNA sequencing (66).

### WES

WES was performed by Novogene in genomic DNA (gDNA) extracted from peripheral blood mononuclear cells. Raw read files were first converted to FASTQ format, then aligned to human genome hg38 using the Burrows-Wheeler aligner (BWA) (67). Aligned reads were processed using Genome Analysis Toolkit (GATK), which also extracted candidate variants/ polymorphisms to reduce sequencing errors (68). Variant annotation was performed by using ANNOVAR (69). A stringent categorization algorithm to avoid false positives was devised, removing: (a) variants with minimum depth of less than 10 or less than 4 reads supporting the alternate allele; (b) synonymous SNVs; (c) variants in repetitive genomic regions. The variant coordinates were crosschecked with the list of somatic mutations in the same patients, and any commonalities were omitted from the germline list.

#### Errors of immunity-linked genes.

We screened this genomic cohort for the presence of rare germline variants associated with primary immunodeficiency in a panel comprised of 464 immune genes defined by the 2022 Updated Classification of Human Inborn Errors of Immunity (IEI) of the IUIS Expert Committee (Supplemental Table 1) (19). Rare variants were defined as those with population allele frequencies of less than 1% obtained from gnomAD. Only variants annotated as missense, nonsense, indel, or splice site were considered for downstream analyses. Each variant was assessed according to the ACMG criteria, using ClinVar (20) and VarSome tools (21). We selected pathogenic/likely pathogenic (P/LP) and VUS overrepresented in our cohort, i.e., with a significant corrected *P* value for the comparison of observed versus expected frequencies according to gnomAD. Multiple-testing correction of *P* values by using Benjamini-Hochberg with a FDR level of 0.05 was applied. Exclusion criteria were: (a) variants with a variant allele frequency (VAF) of less than 40%, (b) variants estimated by the ACMG pathogenicity criteria to be benign or likely benign, and (c) nonoverrepresented VUS, corresponding to a Benjamini-Hochberg FDR above 0.05.

#### T cell lymphoid drivers.

We screened this genomic cohort for the presence of somatic variants in a list of 168 recurrent T cell lymphoid drivers (Supplemental Table 3). The selection of the genes was based on 2 criteria: (a) previously described in T-LGLL according to 2 seminal publications (5, 70); (b) alternatively, not described in T-LGLL but identified as recurrent genes in either mature T cell neoplasms and/or lymphoid clonal hemopoiesis (L-CHIP) (30–33). Missense, nonsense, frameshift, and indel variants were further filtered by pathogenicity criteria according to COSMIC (https://cancer.sanger.ac.uk/cosmic), ClinVar, and VarSome somatic filters. Only P/LP variants were selected to increase stringency in terms of clinical consequences.

Gene-level somatic CNVs were primarily called using CNVkit (71). Values were calculated by mapping genes onto the segment level calls and computing a weighted average along the genomic coordinates. Normalized read depths (log_2_), b-allele frequency (BAF), and CN estimates for ref/alt alleles given the VAF data were extracted. CNVs in hypervariable chromosomic regions (Supplemental Table 13) or CNVs observed in general population datasets (DECIPHER, Database of Genomic Variants [DGV]) (72, 73) were excluded.

### Variant analysis plan

To determine the burden of the rare variants of potential clinical interest in IEI-linked genes, we estimated (a) the individual IEI mutational burden (number of IEI variants per patient); (b) the combined IEI mutational burden in the cohort (proportion of subjects with at least 1 IEI variant); and (c) the simplified expected probability of finding any of the IEI variants in our cohort (sum of individual IEI variant allelic frequencies according to gnomAD). As a control population for statistical comparisons, we estimated the combined mutational burden of the IEI variants found in a cohort of healthy subjects in *All of US* (74). This is a NIH research program aiming to enroll more than one million of US residents aged 18 years or older to create a nationwide population study cohort. Demographics, surveys, clinical information, and biospecimens are donated. To date, short-read whole genome sequencing is available from 245,368 individuals.

The IEI-linked variants included in this work were further clustered and analyzed according to (a) pathogenicity, (b) immune-functional phenotypic implications, and/or (c) pattern of inheritance according to 2022 IUIS Classification of IEI (Supplemental Table 1) (19), and (d) the presumed age period of onset of the associated IEI (i.e., early vs. adult-onset disease) (19, 22). To establish correlations between genomic and biological or clinical data, we further defined a category of hcD variants, considered more likely to predispose to immune misbalance in a carrier, as those being either: (a) P/LP variants, (b) heterozygous VUS for dominant traits, or (c) homozygous/compound heterozygous P/LP/VUS for recessive diseases. Clinical variables, survival outcomes, and laboratory and biological parameters of carriers versus noncarriers of these high-risk variants were compared.

### TCR immunosequencing and analysis

Sequencing of the complementarity determining regions (CDR3) regions of the human TCR β gene was performed using the ImmunoSEQ Assay (Adaptive Biotechnologies), as previously described (45, 75). Deep TCR sequencing data of 145 healthy controls originated from Emerson and DeWitt (original publication and ImmuneACCESS) (43, 44). Downstream analysis of the TCR repertoire was performed exclusively in productive rearrangements (i.e., reads that were in-frame and did not contain a stop codon in their sequence). To overcome intersample differences in depth, downsampling of the TCR repertoires to the optimal threshold of 5,420 clones was performed as a normalization procedure. The diversity metrics calculated per sample included the number of unique clonotypes, unique clone size, and the inverse Simpson index (45) (the lowest value for this index is 1 and the highest value is equal to the number of species). The expansion status of the clones within a repertoire was defined according to the number of templates as (a) nonexpanded (1 template), (b) normally expanded (2–5 templates), (c) pathologically expanded (>5 templates), and (d) hyperexpanded (>10 templates). Condition-related known specificities of the identified clonotypes were annotated according to the dataset from Pagliuca et al. (*n* = 80,220 reference clonotypes, Supplemental Table 12) (45).

### Single-cell RNA+TCRαβ-Seq from T-LGLL and healthy control samples

Preprocessed Seurat objects of scRNA-Seq+ TCRαβ-Seq of flow cytometry–sorted CD45^+^ blood mononuclear cells from T-LGLL samples (*n* = 11) and healthy controls (*n* = 6) from Huuhtanen and Bhattacharya et al., available at https://zenodo.org/records/4739231 (13), were utilized. Clinical characteristics of the samples used are summarized in Supplemental Table 9. Extensive methodological description of this study dataset is available elsewhere (13). We focused our analysis on hyperexpanded T cell clonotypes (>10 TCR templates). Batch-corrected latent embeddings from scVI (version 0.5.0) (76) were used for graph-based clustering and uniform manifold approximation and projection (UMAP) dimensionality reduction implemented in Seurat (v. 3.0.0) with RunUMAP function, and scaled with 3,000 most highly variable genes with the FindVariable function and ScaleData functions with default parameters (77.

### Genomics and transcriptomics from mature T cell cancer cell lines

Genomic data from 26 mature T cell neoplasm cell lines was gathered from The Cancer Dependency Map Project (https://depmap.org, DepMap, Broad Institute) (34). Briefly, DepMap Data Release is a publicly available comprehensive omics resource for understanding cancer biology and identifying potential therapeutic targets. We selected all cell lines matching with the context “Mature T NK cell neoplasms.” Gene-level damaging-supporting SNVs and CN normalized read datasets were analyzed. Bulk RNA-Seq was available for 22 of the 26 cell lines. Read count data from RNA-Seq by expectation-maximization (RSEM) (unstranded mode) was normalized with the Trimmed Mean of M-values (TMM) method in edgeR default option (78). A summary of main biological characteristics and RNA-Seq data used in this study are provided in Supplemental Table 6.

### Gene-expression levels and differential gene-expression analysis

Single-cell and bulk RNA-Seq mean gene-expression levels were compared using *t* test and Wilcoxon’s tests, respectively (79). Differential expression analyses were performed using DESeq2 with default parameters, based on the Wald test with Bonferroni’s correction of *P* values (80). In scRNA-Seq T-LGLL samples, we compared *STAT3*mt versus *STAT3*wt cells. In cell lines with RNA-Seq, we compared *STAT3*mt versus fusion-matched *STAT3*wt cell lines, based on the oncogene fusion present in the *STAT3*mt cells. Enrichment analysis was performed with the list of dysregulated genes (abs log_2_ fold change [log_2_FC] > 0.2; adjusted *P* value [*P*adj] < 0.05) with hypergeometric testing implemented in ClusterProfiler (40) with GO terms gathered from MSigDB (42). Gene-expression scores in scRNA-Seq were calculated with the Seurat AddModuleScore function (41). The TCR score was calculated with 15 genes, including components of the TCR complex (GO:0042101) and the TCR signalosome (GO:0036398). A score with the same set of genes was also calculated in bulk RNA-Seq from the T cell cancer cell lines as geometric means (13). A *STAT3* score, indicative of *STAT3* activation, was also calculated for scRNA-Seq with 9 genes identified by interrogating the set of genes upregulated in *STAT3*mt versus *STAT3*wt T-LGL clones and selecting those genes matching with either a list of upregulated genes in human cells expressing *STAT3* gathered from MSigDB (42) or with the lists of upregulated genes in *STAT3*mt versus *STAT3*wt T cell neoplasm lines from DepMap generated here. To visualize gene expression in scRNA-Seq, scaled expressions were used with the Seurat FeaturePlot function (77). Gene-expression scores were visualized in scRNA-Seq in a similar fashion. Thresholds corresponding to the 90th percentiles of the gene expression scores were set to identify and quantify the proportion of cells with high TCR/STAT3 signaling scores.

### Statistics

Categorical variables were presented as percentages and compared using Pearson’s χ^2^ and Fisher’s exact tests. Continuous variables were presented as mean and SD if normally distributed and as median and IQR if nonnormally distributed.

Differential analysis of categorical variables included Pearson’s χ^2^ or Fisher’s exact tests; comparison of continuous variables included 2-tailed Student’s *t* test or nonparametric Mann-Whitney *U* test. Differences with a 2-tailed *P* values less than 0.05 were considered statistically significant. Survival analysis between groups was done with the log-rank test. Associations between clinical data and survival outcomes were assessed with unadjusted (univariable) and adjusted (multivariable) Cox’s regression. Overall survival estimations were presented with 95% CI.

Gene pathway analysis was performed with using GeneMANIA (University of Toronto, Canada) (81) and Cytoscape (NIH, Bethesda, MD) (82).

Statistical analysis and graphic representation was performed using GraphPad Prism, version 9.4 (GraphPad Software Inc.), and STATA, version 16 (StataCorp LLC) or R (R Core Team, Vienna, Austria) (83). The R packages and functions used for SNVs, CNVs, and scRNA-Seq and bulk RNA-Seq analyses are indicated as per their mention throughout this section.

### Study approval

This research was approved by the institutional review board of Cleveland Clinic. Patient enrollment into clinical registry and specimen collection was performed in accordance with IRB 15-1278 and IRB 5024, respectively. The study was performed in accordance with the Declaration of Helsinki. Written, informed consent was obtained from all participants.

### Data availability

Processed genomic data from patient samples used in this study is available at Zenodo (https://doi.org/10.5281/zenodo.14888890). Processed scRNA+TCR from T-LGLL and healthy control samples is available at Zenodo (https://zenodo.org/records/4739231). Genomic and transcriptomic data from T cell cancer cell lines is available at DepMap (https://depmap.org). All other information, including values of all data points in graphs, is provided in the Supplemental Material and Supporting Data Values files. Additional information not included in the Supplemental Material and Supporting Data Values files is available from the authors upon reasonable request.

## Author contributions

CBP collected and analyzed clinical and molecular data, interpreted results, and wrote the manuscript. MW and ZB performed sample processing. CG, NK, AM, LG, CH, NDW, SU, MO, AA, and OO collected data and edited the manuscript. JH and SM generated and provided scRNA-Seq data for analysis and edited the manuscript. JH, YK, SP, and AD analyzed molecular data and edited the manuscript. CG, VV, and JPM provided invaluable help to the manuscript preparation, generated and conceived the study design, designed figures and tables, and wrote the manuscript. All authors participated in the critical review of the final paper and submission.

## Supplementary Material

Supplemental data

ICMJE disclosure forms

Supplemental tables 1-13

Supporting data values

## Figures and Tables

**Table 2:**
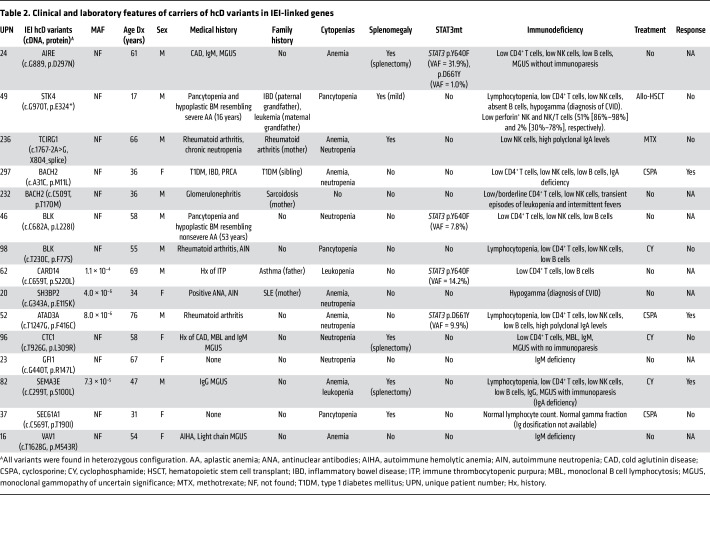
Clinical and laboratory features of carriers of hcD variants in IEI-linked genes

**Table 1:**
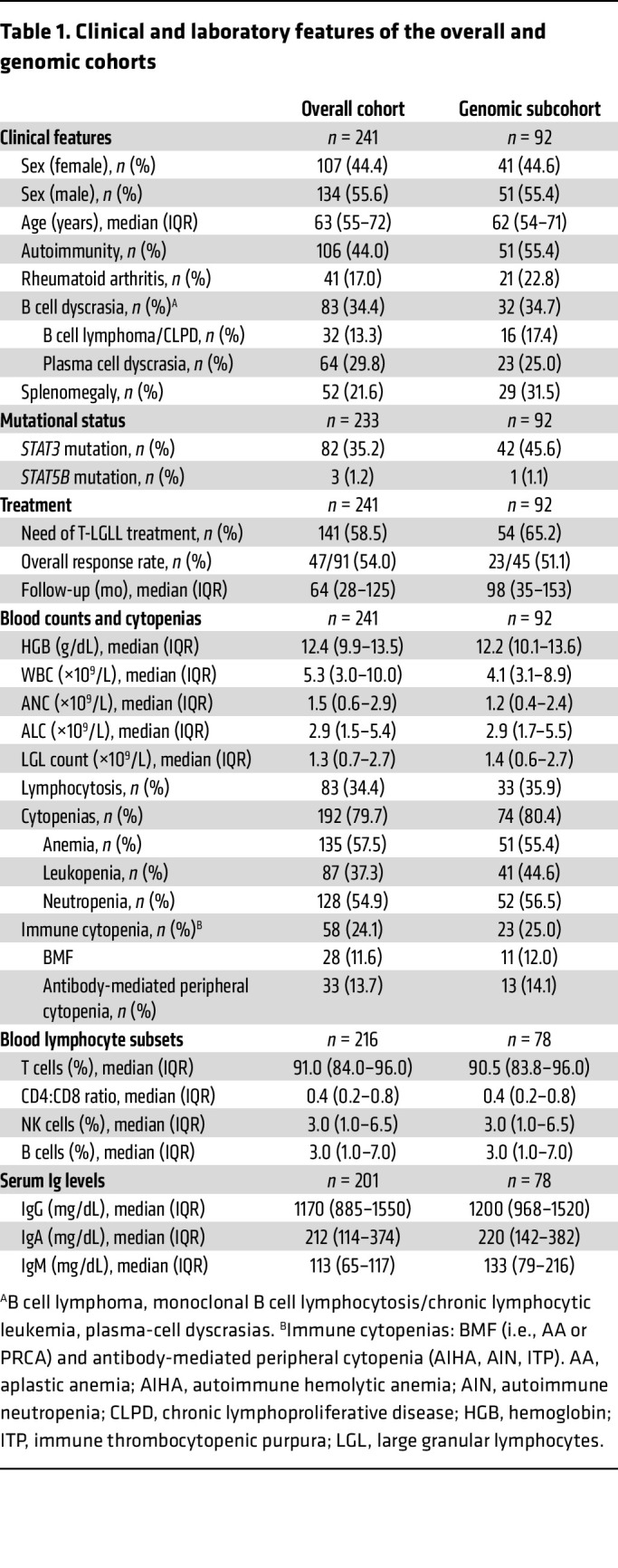
Clinical and laboratory features of the overall and genomic cohorts

## Version 1 05/01/2025

Electronic publication
