# Supplementary material for: Inborn errors of immunity underlie clonal T cell expansions in large granular lymphocyte leukemia
Source: J Clin Invest. 2025 May 1;135(9):e184431. doi: 10.1172/JCI184431 (PMC12043085; doi:10.1172/JCI184431)
Supplement: Supplemental data [file jci-135-184431-s104.pdf]

Bravo-Perez et al. 2025. Inborn errors of immunity underlie clonal T-cell expansions in large granular lymphocyte leukemia

Supplemental Material

Supplemental Methods

Clinical cohort……………………………………………………………………………………………3
Targeted sequencing ………………………………………………………………………………………4
Whole exome sequencing ……………………………………………………………………………5
Variant annotation …………………………………………………………………………………………5
Variant analysis plan………………………………………………………………………………………8
TCR immunosequencing ……………………………………………………………………………………9
Single-cell RNA+TCRαβ in T-LGLL and healthy control samples . ……………………………………10
Mature T-cell neoplasm datasets …………………………………………………………………………11
Gene expression analysis…………………………………………………………………………………11
Statistics ……………………………………………………………………………………………………13

Supplemental Tables and Figures

Supplemental Tables 1-13 (legends only) ………………………………………………………………13
Supplemental Figure 1 ……………………………………………………………………………………16
Supplemental Figure 2 ……………………………………………………………………………………18
Supplemental Figure 3 ……………………………………………………………………………………20
Supplemental Figure 4 ……………………………………………………………………………………22
Supplemental Figure 5 ……………………………………………………………………………………24
Supplemental Figure 6 ……………………………………………………………………………………26
Supplemental Figure 7 ……………………………………………………………………………………28
Supplemental Figure 8 ……………………………………………………………………………………30
Supplemental Figure 9 ……………………………………………………………………………………32
Supplemental Figure 10. …………………………………………………………………………………34
Supplemental Figure 11 …………………………………………………………………………………36

Supplemental Figure 12 ……………………………………………………………………………………38
Supplemental Figure 13……………………………………………………………………………………40
Supplemental Figure 14……………………………………………………………………………………42
Supplemental Figure 15……………………………………………………………………………………44

References

References……………………………………………………………………………………………46

Supplemental Methods

Sex as a biological variable

This study examined male and female participants, as both men and women were eligible, and findings were similar for both sexes.

Clinical cohort

This cross-sectional, genetic association study was performed in a cohort of consecutive patients with T-cell large granular lymphocytosis (T-LGLL) diagnosed and managed at Taussig Cancer Center, Cleveland Clinic Foundation from 1998 to 2023, described elsewhere (**Table 1**)(1). Briefly, diagnosis of T-LGLL required the presence of >4/6 criteria: i) LGLs (>0.40 × 10^9/L) in blood for more than 6 months; (ii) abnormal CTL expressing CD2, CD56 and CD57 and lacking CD28; (iii) preferential usage of a T-cell receptor (TCR) Vb family by flow cytometry; (iv) TCR gene rearrangement by PCR; (v) *STAT3/5B* mutation; (vi) T-LGL infiltration of the bone marrow(2). Cases with NK-LGLL were excluded from this study because our focus on clonal T-cell expansions, as well as because NK-LGLL constitute a less frequent and less defined clinical/molecular variant of the disease.

Clinical data collected comprised patient demographics; presence of splenomegaly; associated autoimmune conditions; bone marrow failure (i.e., pure red cell aplasia [PRCA], aplastic anemia [AA]); antibody-mediated peripheral autoimmune cytopenias (i.e., autoimmune hemolytic anemia [AIHA], autoimmune neutropenia [AIN], primary immune thrombocytopenia [ITP]); personal history of solid and hematological neoplasms, and family history of immune disorders or hemato-lymphoid neoplasms. The presence of a B-cell dyscrasia was considered with the diagnosis of any of the following conditions: monoclonal B-cell lymphocytosis (MBL), chronic lymphocytic leukemia (CLL) or other chronic B-cell lymphoproliferative disorders, Hodgkin lymphoma, Non-Hodgkin B-cell lymphoma, monoclonal gammopathy of undetermined significance (MGUS), myeloma or other plasma cell dyscrasias(3). Acquired causes potentially leading to hypogammaglobulinemia, described elsewhere(4), were

screened in patient's medical charts prior to T-LGLL diagnosis, including: history of B-cell dyscrasias; the use of therapies against B cells or plasma cells; diagnosis of myeloid neoplasms; the use of chemotherapy for hematologic or solid neoplasms; the use of other immunosuppressive therapies; history of solid transplant or hematopoietic stem cell transplant; diagnosis of common variable immunodeficiency (CVID) or other immunodeficiency syndrome; and diagnosis of thymoma/Good syndrome. Overall survival (OS) was defined as time to death from diagnosis. Event-free survival (EFS) was defined by the occurrence of treatment initiation, transfusion dependency, splenectomy, hematopoietic stem cell transplant, transformation to high-grade lymphoma or death.

Laboratory data at diagnosis comprised complete blood count, LGL count, immunoglobulin (Ig) levels, and M protein assessment. Lymphocyte subset characterization by flow cytometry (in $CD45^{Ly}$ gated cells) routinely performed in the clinic in peripheral blood at LGL diagnosis was also collected for the study of the following populations: T cells (CD3+, normal range [NR]: 0.96 - 2.39 $x10^9$/L), CD4 T helper cells (CD3+CD4+, NR: 0.53 - 1.67 $x10^9$/L), CD8 cytotoxic T lymphocytes (CTLs, CD3+CD8+, NR: 0.28 - 0.96 $x10^9$/L), NK cells (CD3-CD16/CD56+, NR: 0.10 - 0.57 $x10^9$/L) and B cells (CD19+, NR: 0.08 - 0.66 $x10^9$/L). The Ig levels analyzed included the quantification of IgG (NR: 717 - 1411 mg/dL), IgA (NR: 78 - 391 mg/dL) and IgM (NR: 53 - 334 mg/dL).

Targeted sequencing

All patients were deep sequenced for the presence of a mutation in exon 21 of *STAT3*, the protein-protein interaction domain as previously described(5).

Targeted sequencing was performed as previously described using a custom panel for detection of hematological neoplasm gene variants from TruSeq or Nextera platforms (Illumina, San Diego, CA)(6, 7). Nucleic acid extracted from the specimen was subjected to nested multiplex PCR-based target enrichment. Coding and non-coding regions of 63 genes were amplified and sequenced on an Illumina instrument with paired end, 150x2 cycle reads. A customized bioinformatic analytical pipeline was used

to map reads to human genome hg38. During internal validation, this test delivered an average of >500X coverage and >98% of targeted regions showed over 100X coverage. The test demonstrated 95.2% sensitivity and 99.9% specificity in identifying single nucleotide variants (SNVs), small insertions and deletions (indels) (<10bp) of >5% variant allele fraction (VAF). For the identification of large indels (>10bp) >5% VAF, including FLT3 ITD, the test demonstrated 87.5% sensitivity and 99.9% specificity. The limit of detection of this test is 1% for the JAK2 V617F and NPM1 W288Cfs*12 variants, and 5% for other variants.

Whole exome sequencing, SNVs and CNVs calling and quality filtering

Whole exome sequencing (WES) was performed by Novogene (Sacramento, CA) in genomic DNA (gDNA) extracted from peripheral blood mononuclear cells. Briefly, the gDNA was randomly sheared into short fragments (180-280 bp). The obtained fragments were end repaired, A-tailed, and further ligated with Illumina adapters. The fragments with adapters were PCR amplified, size selected, and purified. The prepped libraries were hybridized in the buffer with biotin-labeled probes, and magnetic beads with streptavidin were used to capture the exons of genes. Subsequently, non-hybridized fragments were washed out and probes were digested. The captured libraries were enriched by PCR amplification. The library was checked with Qubit and real-time PCR for quantification and bioanalyzer for size distribution detection. Quantified libraries were pooled and sequenced on Illumina platforms with PE150 strategy, according to effective library concentration and data amount required.

Raw read files were first converted to FASTQ format, then aligned to human genome hg38 using the Burrows-Wheeler Aligner (BWA) (8). Aligned reads were processed using Genome Analysis Toolkit (GATK) that also extracted candidate variants/ polymorphisms to reduce sequencing errors (9).

Variant annotation and variant/gene/patient filtering

Variant annotation was performed by using ANNOVAR (10). A stringent categorization algorithm to

avoid false positives was devised, removing: (i) variants with minimum depth <10 or <4 reads supporting the alternate allele; (ii) synonymous SNVs; (iii) variants in repetitive genomic regions. The variant coordinates were crosschecked with the list of somatic mutations in the same patients, and any commonalities were omitted from the germline list.

Errors of immunity-linked genes

We screened this study cohort for the presence of rare germline variants associated with primary immunodeficiency in a panel comprised by 464 immune genes defined by the 2022 Updated Classification of Human inborn errors of immunity (IEI) of the IUIS Expert Committee (**Supplemental Table 1**) (11). Rare variants were defined as those with population allele frequencies below 1% obtained from the Genome Aggregation Database (gnomAD). Only variants annotated as missense, nonsense, indel, or splice site were considered for downstream analyses. Each variant was assessed according to the American College of Medical Genetics (ACMG) criteria, using ClinVar (12) and VarSome tools (13). Prediction scores for the effect of gene variants in protein function or structure SIFT (14), PROVEAN (15), LRT (16), MutationTaster (17), MutationAssessor (18), FATHMM (19), and CADD (20), were additionally conducted. For splice site variants, Mutation Taster, CADD and MaxEntScan scores (21) were used. For the purpose of this study, we selected all pathogenic/likely pathogenic (P/LP) and variants of uncertain significance (VUS) overrepresented in our cohort, i.e. with a significant corrected p-value for the comparison of observed vs. expected frequencies according to gnomAD. We performed a binomial test against the null hypothesis that the alternate allele was drawn from a binomial (*n,f*) distribution, where *n* was the allele frequency observed in our cohort and *f* was the expected frequency (MAF) in gnomAD. Multiple-testing correction of p-values by using Benjamini-Hochberg with a false discovery rate (FDR) level of 0.05 was applied. Exclusion criteria were: i) variants with a VAF<40%, ii) variants estimated by the ACMG pathogenicity criteria to be benign or likely benign, and iii) non-overrepresented VUS, corresponding to a Benjamini-Hochberg FDR above 0.05.

T-cell lymphoid drivers

We screened this genomic study cohort for the presence of somatic variants in a list of 168 recurrent T-cell lymphoid drivers (Supplemental Table 3). The selection of the genes was based on two criteria: (i) previously described in LGLL according to two seminal publications (22, 23); (ii) alternatively, not described in LGLL but identified as recurrent genes in either mature T-cell neoplasms and/or lymphoid clonal hemopoiesis (L-CHIP)(24–27). For the latter case, the lists of mutated genes in mature T-cell lymphoid neoplasms and of L-CHIP genes were cross-matched, and we incorporated into the final panel all recurrent (>1 study) genes in the first dataset and positive matches between T-cell lymphoma/leukemia and L-CHIP genes from the second dataset -therefore, CHIP genes restricted to myeloid or B-cell lymphoid neoplasms were excluded. In-parallel run targeted sequencing for a hematological cancer NGS panel (TruSeq or Nextera platforms, Illumina, San Diego, CA) that was also interrogated for the overlapping genes to increase diagnostic yield. Detected variants were filtered out using the following exclusion criteria: (i) minimum depth below of 10 reads or less than 4 reads supporting the alternate allele; (ii) synonymous variants, (iii) polymorphisms (global population frequency >1%), and potential germline variants. Missense, nonsense, frameshift, and indels variants fulfilling the selection criteria were further filtered by pathogenicity criteria according to COSMIC, ClinVar and VarSome with somatic filters. Only P/LP variants registered in COSMIC as canonical/recurrent and ever reported as somatic were selected to increase stringency in terms of clinical consequences.

Gene-level somatic copy number variants (CNVs) were primarily called using CNVkit(28). Values were calculated by mapping genes onto the segment level calls and computing a weighted average along the genomic coordinates. Normalized read depths (log2), b-allele frequency (BAF), and CN estimates for ref/alt alleles given the VAF data we extracted. CNVs in hypervariable chromosomic regions (**Supplemental Table 11**), or CNVs observed in general population datasets (DECIPHER, Database of Genomic Variants [DGV])(29, 30) were excluded.

Variant analysis plan

To determine the burden of the rare variants of potential clinical interest in IEI-linked genes, we estimated for the P/LP and VUS variants found to be overrepresented in our cohort: (i) the individual IEI mutational burden, defined as the number of IEI variants of per patient; (ii) the combined IEI mutational burden in the cohort, calculated as the proportion (%) of subjects with at least one of the IEI variants; and (iii) the simplified expected probability of finding any of the IEI variants in our cohort, estimated as the sum of f_i, where f_i was the allelic frequency expected for each variant according to gnomAD (used here as a comparator extrapolated from the general population).

As a control population for statistical comparisons, we estimated the combined mutational burden of the variants found in a cohort of healthy subjects in *All of US* (31). This is a National Institutes of Health (NIH) research program aiming to enroll more than one million of US residents aged ≥18 years to create a nationwide population study cohort. Demographics, surveys, clinical information, and bio-specimens are donated. The *All of Us* program stores diagnosis codes harmonized into the SNOMED clinical terms vocabulary. To date, short-read whole genome sequencing is available from 245,368 individuals. As healthy control population to test the presence of the variants found, we considered cases with: (i) none of the 36,920 SNOMED terms coding any clinical condition, and (ii) single-nucleotide/indel variant information available from short-read sequencing.

The IEI-linked variants included in this work were further clustered and analyzed according to: (i) pathogenicity, (ii) immune functional-phenotypic implications and/or (iii) pattern of inheritance according to 2022 IUIS Classification of IEI (**Supplemental Table 1**) (11), and (iv) the presumed age period of onset of the associated IEI (i.e., early- vs. adult-onset disease) (11, 32). To establish correlations between genomic and biological or clinical data, we further defined a category of high-confidence deleterious (hcD) variants, considered more likely to predispose to immune misbalance in a carrier, as those being either: (i) P/LP variants, (ii) heterozygous VUS for dominant traits, or (iii) homozygous/compound heterozygous P/LP/VUS for recessive diseases. Clinical variables, survival

outcomes, and laboratory and biological parameters of carriers vs. non-carriers of these high-risk variants were compared.

TCR immunosequencing and analysis

Sequencing of the complementarity determining regions (CDR3) regions of human T-cell receptor (TCR) beta gene was performed using the ImmunoSEQ Assay (Adaptive Biotechnologies), as previously described (33, 34). Briefly, gDNA extracted from peripheral blood mononuclear cells was amplified in a bias-controlled multiplex PCR, with a first PCR consisting of forward and reverse primers specific for every V and J gene segment allowing the amplification of the hypervariable CDR3 region, and a second PCR by which a proprietary barcode sequence and Illumina adapters were added. CDR3 libraries were sequenced on Illumina platforms. Deep TCR sequencing data of 145 healthy controls originated from Emerson and DeWitt (original publication and ImmuneACCESS) (35, 36). The immunoSEQ Analyzer 3.0 software (Adaptive Biotechnologies) was used for sample data export, preliminary statistics and quality control steps. Annotation was in accordance with the IMGT database (https://www.imgt.org).

Downstream analysis of the TCR repertoire was performed exclusively in productive rearrangements (i.e., translating a functional amino acid sequence, intended as reads that were in-frame and did not contain a stop codon in their sequence). Down-sampling, a normalization procedure of resampling the TCR repertoire for all the specimens to the optimally minimal depth of the samples sequenced, was done to overcome the issue related to inter-sample differences in depth. An optimal repertoire size threshold of 5420 clones was used for down-sampling in both cohorts. The diversity metrics calculated per sample included: the number of unique clonotypes, unique clone size, and the inverse Simpson index, $ISI = (\sum^{T}_{i=1} p^{2}_{i})^{-1}$, where p_i is the proportional abundance of each unique clonotype and T is the total number of unique clonotypes (the lowest value for this index is 1 and the highest value is equal to the number of species). The expansion status of the clones within a repertoire was defined according

to the number of templates and the clonotypic distribution in healthy control as: (i) non-expanded (1 template), (ii) normally expanded (2-5 templates), (iii) pathologically expanded (>5 templates), and hyperexpanded clonotypes (>10 templates). Condition-related known specificities of the identified clonotypes were annotated according to the dataset from Pagliuca et al. (80,220 references, **Supplemental Table 10**), which included, among others, human TCR beta sequences from the public databases VDJDB (https://vdjdb.cdr3.net/search), McPAS-TCR (http://friedmanlab.weizmann.ac.il/McPAS-TCR/), and PIRD TBAdb (https://db.cngb.org/pird/tbadb/) (34).

Single-cell RNA+TCRαβ-seq from T-LGLL and healthy control samples

Preprocessed Seurat objects of scRNA+TCRαβ-seq of flow cytometry-sorted CD45+ blood mononuclear cells from T-LGLL (n=11) and healthy control (n=6) samples, independently repurposed from study by Huuhtanen and Bhattacharya et al., available at https://zenodo.org/records/4739231, were utilized (37). Clinical characteristics of the samples used are summarized in **Supplemental Table 8**. Extensive methodological description of this dataset is available in the published paper (37). We focused our analysis on hyperexpanded T-cell clonotypes (>10 TCR templates). Batch-corrected latent embeddings from scVI (v.0.5.0) were used for graph-based clustering and uniform manifold approximation and projection (UMAP) dimensionality reduction implemented in Seurat (v.3.0.0) with RunUMAP function, and scaled with 3,000 most highly variable genes with the FindVariable function and ScaleData functions with default parameters (38, 39).

Genomics and transcriptomics from mature T-cell cancer cell lines

Genomic data from 26 mature T-cell neoplasm cell lines was gathered from The Cancer Dependency Map Project (https://depmap.org, DepMap, Broad Institute) (40). Briefly, DepMap Data Release is a publicly available comprehensive omics resource for understanding cancer biology and identifying potential therapeutic targets. We selected all cell lines matching with the context “Mature T NK cell

neoplasms". Gene-level damaging-supporting SNVs and CN normalized read datasets were analyzed. Bulk RNA-seq was available for 22 of the 26 cell lines. Read count data from RSEM (unstranded mode) was normalized with the Trimmed Mean of M-values (TMM) method in edgeR default option(41). A summary of main biological characteristics, and RNA-seq data used in this study is provided in **Supplemental Table 9**.

Gene expression levels and differential gene expression analysis

Single-cell and bulk RNA-seq mean gene expression levels were compared using t-test and Wilcoxon tests, respectively (42). Differential expression analyses were performed using DESeq2 with default parameters, based on the Wald test with Bonferroni correction of p-values (43). In scRNAseq T-LGLL samples, we compared *STAT3*mt vs. *STAT3*wt cells. In cell lines with RNA-seq, we compared *STAT3*mt vs. fusion-matched *STAT3*wt cell lines, based on the oncogene fusion present in the *STAT3*mt cells. Three cell lines had amplification of *STAT3*: SMZ1 and SUDHL1, with rearrangements in TP63 and ALK, and OCILY12, without a driver oncogene fusion. Therefore, three sets of DE analysis were set: i) SMZ1 vs. TP63-rearranged, *STAT3*wt cells; ii) SUDHL1 vs. ALK-rearranged, *STAT3*wt cells; iii) OCILY12 vs. non-rearranged *STAT3*wt cells. Enrichment Gene Ontology (GO) pathway analysis was performed with the list of dysregulated genes (abs(log2FC)>0.2; padj<0.05) using the enrichGO function implemented in ClusterProfiler with Benjamini–Yekutieli correction and FDR<0.10 (44). Gene expression scores in scRNA-seq were calculated with the Seurat AddModuleScore function (45). The TCR score was calculated with 15 genes (*TRAC, TRDC, CD2, CD3D, CD3E, CD3G, CD247*, *CD4, CD5, CD6, CD8A, SYK, ZAP70, LCK, LAT*), including components of the TCR complex (GO:0042101) and the TCR signalosome (GO:0036398). A score with the same set of genes was also calculated in bulk-RNA seq from the T-cell cancer cell lines as geometric means(37). A *STAT3* score, indicative of *STAT3* activation, was also calculated for scRNA-seq with 9 genes *(STAT3, TNFSF9, CCL3, GSTP1, PECAM1, CTSD, NKG7, BCL3, MYADM*). The genes were selected by interrogating the set of genes

upregulated in *STAT3*mt vs. *STAT3*wt T-LGL clones and selecting those genes matching with either a list of upregulated genes in human cells expressing *STAT3* off-a-viral-vector gathered from MSigDB (DAUER_*STAT3*_TARGETS_UP)(46), or with the lists of upregulated genes in *STAT3*mt vs. *STAT3*wt T-cell neoplasm lines from DepMap generated here. To visualize gene expression in scRNA-seq, scaled expressions were used with the Seurat FeaturePlot function(39). Gene expression scores were visualized in scRNA-seq in a similar fashion. Thresholds corresponding to the 90th percentiles of the gene expression scores were set to identify and quantify the proportion of cells with high TCR/STAT3 signaling scores.

Statistics

Categorical variables were presented as percentages and compared using Pearson's chi-squared and Fisher's exact tests. Continuous variables were presented as mean and SD if normally distributed, and as median and IQR if non-normally distributed.

Differential analysis of categorical variables included Pearson's $\chi 2$ or Fisher's exact tests; comparison of continuous variables included Student's t test or nonparametric Mann-Whitney U test. Differences with a 2-tailed p-values less than 0.05 were considered statistically significant. Survival analysis between groups was done with Log-Rank test. Associations between clinical data and survival outcomes were assessed with unadjusted (univariable) and adjusted (multivariable) Cox regression. Overall survival Estimations were presented with 95% confidence intervals (95%CI).

Mutation gene pathway analysis was performed with using GeneMANIA (University of Toronto, Canada)(47) and Cytoscape (NIH, Bethesda, MD) (48).

Statistical analysis and graphic representation was performed using GraphPad Prism v.9.4 (GraphPad Software Inc., San Diego, CA), STATA v.16 (StataCorp LLC, College Station, TX), or R (R Core Team, Vienna, Austria)(49). The R packages and functions used for SNVs, CNVs and scRNA-seq and bulk-RNA-seq analyses are indicated as per their mention throughout this section.

Supplemental Tables

Supplemental Table 1 (Excel File). Panel of genes associated with IEI screened in this study. The list of 464 genes is adapted from 2022 IUIS Classification of IEI adapted from: Bousfiha AM et al. J Clin Immunol. 2022 Oct;42(7):1473-1507. Disease name, pattern of inheritance and OMIM# are provided. Abbreviations: AD: autosomal dominant, AR: autosomal recessive, DN: dominant-negative, GOF: gain of function, LOF: loss of function, XL: X-linked.

Supplemental Table 2 (Excel File). List of gene variants associated with IEI identified in this study. Information regarding OMIM#, gene variant chromosome and genomic position (hg19), nucleotide and amino acid change, gnomAD population frequency, pathogenicity prediction according to SIFT, PROVEAN, LRT, MutationTaster, MutationAssessor, FATHMM and CADD and classification according to American College of Medical Genetics (ACMG) criteria is provided. Abbreviations: B: benign, BM: benign moderate, BS: benign supporting/strong, LB: likely benign, LP: likely pathogenic, U: uncertain, P: pathogenic, PM: pathogenic moderate, PS: pathogenic supporting/strong. VUS: variant of uncertain significance.

Supplemental Table 3 (Excel File). Survival analysis - multivariate Cox regression. †Defined as the initiation of therapy, need of transfusions, splenectomy, transplant, transformation to high-grade lymphoma, or death. hcD: high-confidence deleterious variants in IEI-linked genes. HR: hazard ratio. EFS: progression free survival. OS: overall survival. 95%CI: 95% confidence interval.

Supplemental Table 4 (Excel File). Panel of putative lymphoid driver genes screened in this study. The list includes genes reported as recurrently mutated in LGLL, mature T-cell lymphoid neoplasms, and lymphoid clonal hemopoiesis (L-CHIP). Ensembl annotations are provided.

Supplemental Table 5 (Excel File). List of mutations in T-cell lymphoid drivers in the WES cohort. Information regarding variant cDNA and protein changes (human genome hg38), and VAF is provided. Abbreviations: VAF: variant allele frequency.

Supplemental Table 6 (Excel File). Summary of mature T-cell neoplasm cell lines gathered from DepMap. Full information on this dataset is available in: https://depmap.org/portal/. Neoplasia subtype, ploidy, oncogenic fusions, availability of WES and RNA-seq, *STAT3* amplification status estimated as described in Supplementary Methods, and Set# for differential expression analysis are provided. Abbreviations: ALCL: Anaplastic large cell lymphoma. CTCL: cutaneous T cell lymphoma. ATLL: adult T-cell leukemia/lymphoma. HSTCL: hepatosplenic T-cell lymphoma. T-LGLL: T-cell large granular lymphocytic leukemia. PTCL: peripheral T cell lymphoma. Ploidy is abbreviated as 2n: near-diploid, 2n+: hyperdiploid, 3n: triploid, 4n: tetraploid.

Supplemental Table 7 (Excel File). Copy number variants (CNVs) in T-LGLL patients with WES data. Chromosome position (human genome hg38), chromosomic band, gene(s) involved and CNV type are provided.

Supplemental Table 8 (Excel File). Summary results of gene pathway analysis for the high-confidence deleterious variants (hcD) in IEI genes and genes dysregulated in T-LGLL. The enriched pathways are listed in three columns: (i) only in IEI network, (ii) only in T-LGLL network and (iii) common pathways. GO annotation is used. Abbreviations: hcD: high-confidence deleterious variants, IEI: inborn errors of immunity, GO: gene ontology, T-LGLL: T-cell large granular lymphocytic leukemia.

Supplemental Table 9 (Excel File). Summary of clinical characteristics of scRNA-seq T-LGLL and healthy control samples. Full information on this dataset is available in: Huuhtanen J et al. Nat Commun. 2022. 13:1981. PMID: 35411050. Abbreviations: Pt: patient. HC: healthy control.

Supplemental Table 10 (Excel File). Differentially expressed genes between STAT3mt and STAT3wt hyperexpanded T-cells from T-LGLL samples (n=11). Average log2FoldChange (avg_logFC) values > 0 correspond to genes upregulated in STAT3mt (i.e. downregulated in STAT3wt) T-LGL clones; avg_logFC < 0 correspond to genes downregulated in STAT3mt (upregulated in STAT3wt) T-LGL clones. Full information on this dataset is available in: Huuhtanen J et al. Nat Commun. 2022. 13:1981. PMID: 35411050.

Supplemental Table 11 (Excel File). Differentially expressed genes between STAT3mt and STAT3wt fusion-matched T-cell cancer cell lines from DepMap. Three fusion-matched comparison sets were defined, based on the oncogene fusion present in the STAT3mt cells (see Supplemental Table 6). Average log2FoldChange (avg_logFC) values > 0 correspond to genes upregulated in STAT3mt (i.e. downregulated in STAT3wt) cell lines; avg_logFC < 0 correspond to genes downregulated in STAT3mt (upregulated in STAT3wt) cell lines.

Supplemental Table 12 (Excel File). Meta-analytic dataset of CDR3 sequences. Modified from Pagliuca S et al. JCI Insight. 2021 Jul 8;6(13):e149080.

Supplemental Table 13 (Excel File). Coordinates of hypervariable chromosomic regions. Reference human genome hg38.

Supplementary Figures

Supplemental Figure 1. Mutations in genes with hcD variants identified in T-LGLL patients (1): *AIRE, ATAD3A, BACH2, BLK, CARD14, CTC1*. Annotation of the domains of the proteins coded by the canonical transcripts was extracted from Ensembl and UniProt .json files. The mutations labelled in red with the amino acid change are the ones found in our study. The plot additionally displays rare (MAF<1%) deleterious variants previously reported in these genes using gnomAD genomic browser v4.1.0, integrating pathogenicity predictors and variant frequency (number of variants reported in gnomAD). GOF: Gain-of-function. LOF/pLOF: Loss of function/predicted LOF. NOS: region/domain not otherwise specified.

Suppl. Fig.1

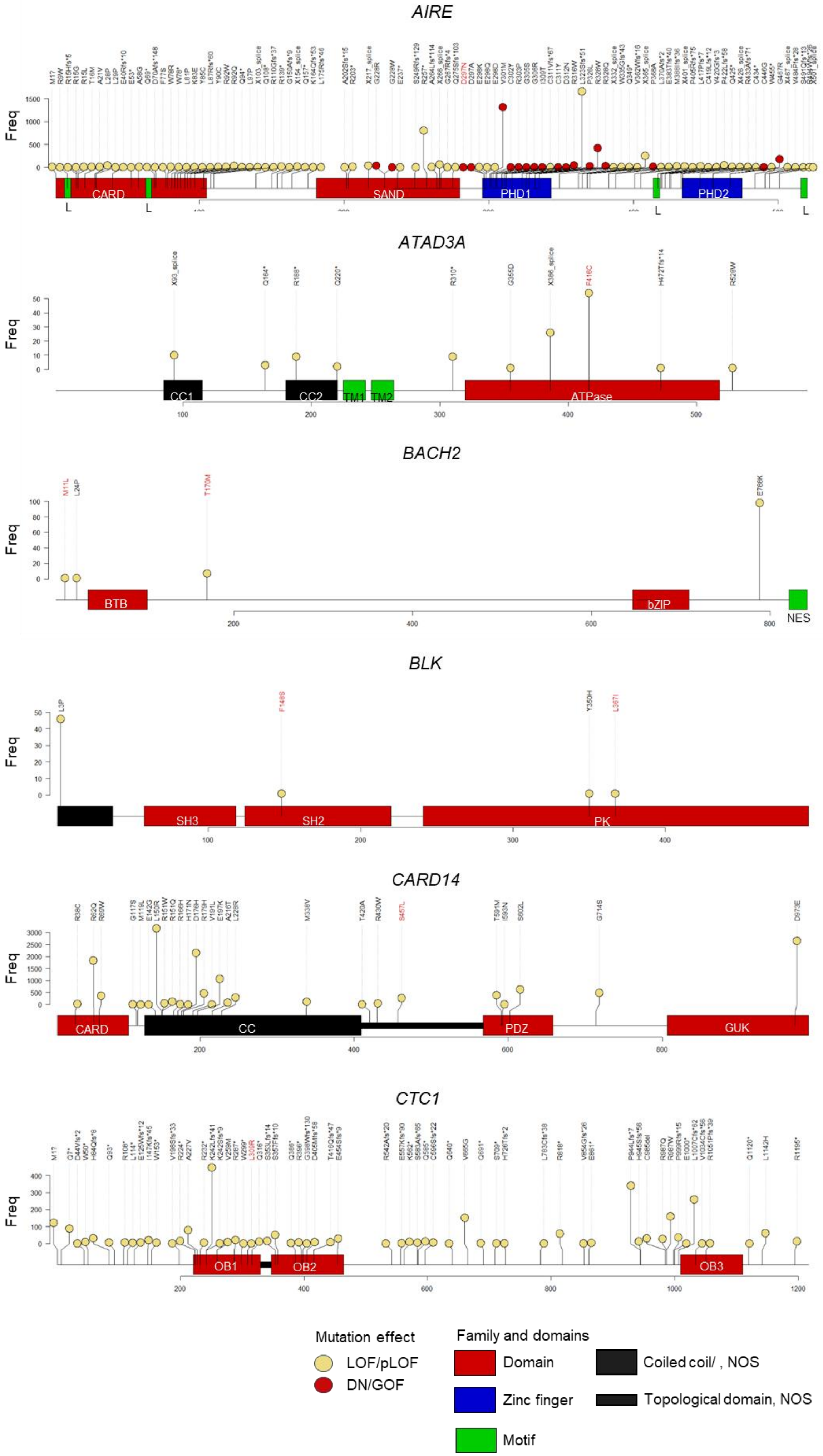
AIRE
Freq
CARD
SAND
PHD1
PHD2
L
ATAD3A
CC1
CC2
TM1
TM2
ATPase
BACH2
BTB
bZIP
NES
BLK
SH3
SH2
PK
CARD14
CARD
CC
PDZ
GUK
CTC1
OB1
OB2
OB3
Mutation effect
LOF/pLOF
DN/GOF
Family and domains
Domain
Zinc finger
Motif
Coiled coil/ , NOS
Topological domain, NOS

Supplemental Figure 2. Mutations in genes with hcD variants identified in T-LGLL patients (2): ***GFI1, SEC61A1, SEMAE, SH3BP2, STK4, VAV1***. Annotation of the domains of the proteins coded by the canonical transcripts was extracted from Ensembl and UniProt .json files. The mutations labelled in red with the amino acid change are the ones found in our study. The plot additionally displays rare (MAF<1%) deleterious variants previously reported in these genes using gnomAD genomic browser v4.1.0, integrating pathogenicity predictors and variant frequency (number of variants reported in gnomAD). GOF: Gain-of-function. LOF/pLOF: Loss of function/predicted LOF. NOS: region/domain not otherwise specified.

Suppl. Fig.2

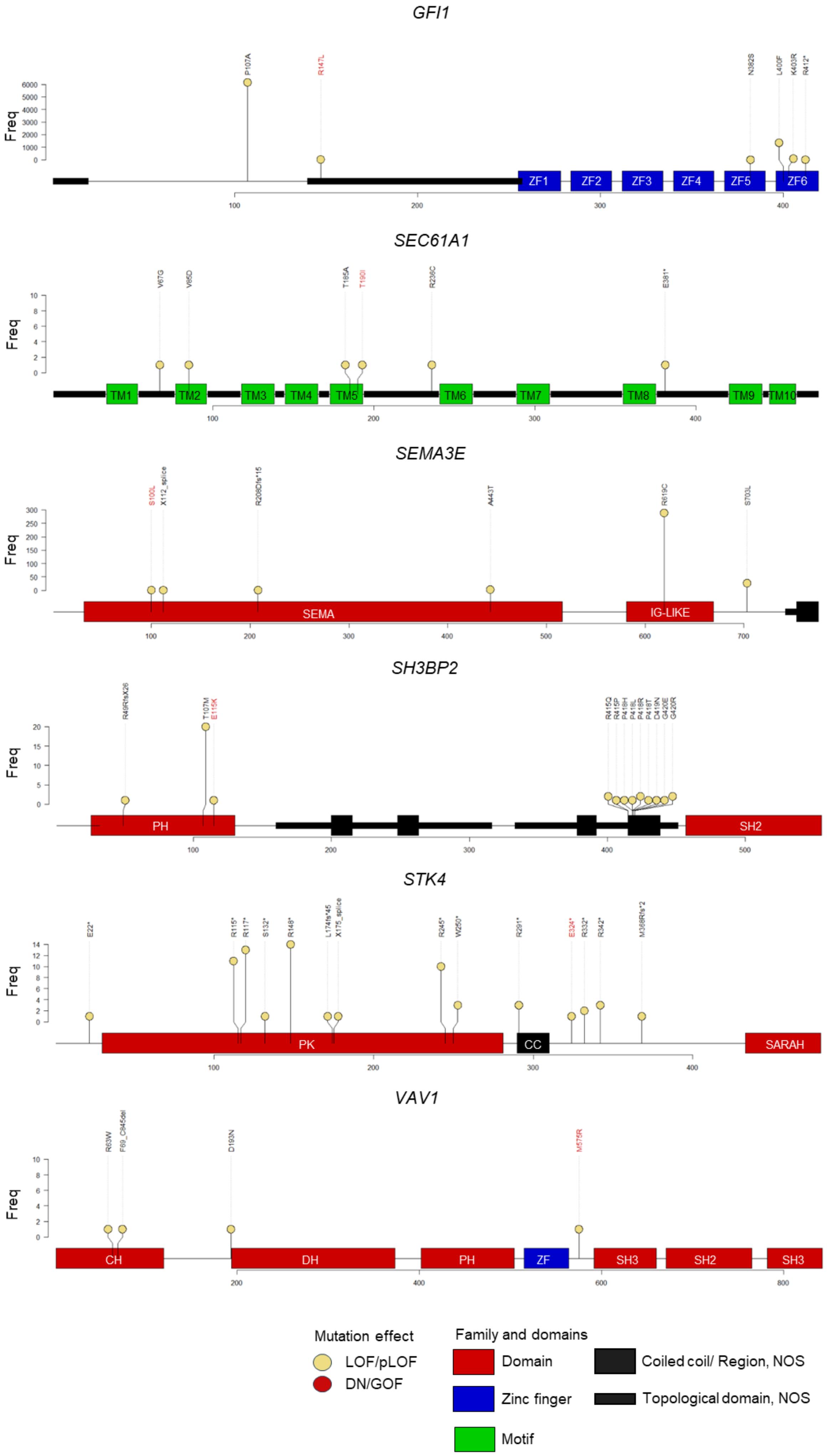
GFI1
Freq
P107A
R147L
N382S
L400F
K403R
R412*
ZF1
ZF2
ZF3
ZF4
ZF5
ZF6
SEC61A1
Freq
V67G
V85D
T185A
T190I
R236C
E381*
TM1
TM2
TM3
TM4
TM5
TM6
TM7
TM8
TM9
TM10
SEMA3E
Freq
S100L
X112_splice
R208Dfs*15
A443T
R619C
S703L
SEMA
IG-LIKE
SH3BP2
Freq
R49RfsX26
T107M
E115K
R415Q
R415P
P418H
P418L
P418R
P418T
D419N
G420E
G420R
PH
SH2
STK4
Freq
E22*
R115*
R117*
S132*
R148*
L174fs*45
X175_splice
R245*
W250*
R291*
E324*
R332*
R342*
M368Rfs*2
PK
CC
SARAH
VAV1
Freq
R63W
F69_C845del
D193N
M575R
CH
DH
PH
ZF
SH3
SH2
SH3
Mutation effect
LOF/pLOF
DN/GOF
Family and domains
Domain
Zinc finger
Motif
Coiled coil/ Region, NOS
Topological domain, NOS

Supplemental Figure 3. Mutations in IEI genes with hcD variants identified in T-LGLL patients (3): *TCIRG1*. Annotation of the domains of the proteins coded by the canonical transcripts was extracted from Ensembl and UniProt .json files. Exon-protein correlations for both the canonical TCIRG1 and alternative TIRC7 transcripts are shown. The mutations labelled in red with the amino acid change are the ones found in our study. The plot additionally displays rare (MAF<1%) deleterious variants previously reported in these genes using gnomAD genomic browser v4.1.0, integrating pathogenicity predictors and variant frequency (number of variants reported in gnomAD). GOF: Gain-of-function. LOF/pLOF: Loss of function/predicted LOF. NOS: region/domain not otherwise specified.

Suppl. Fig.3

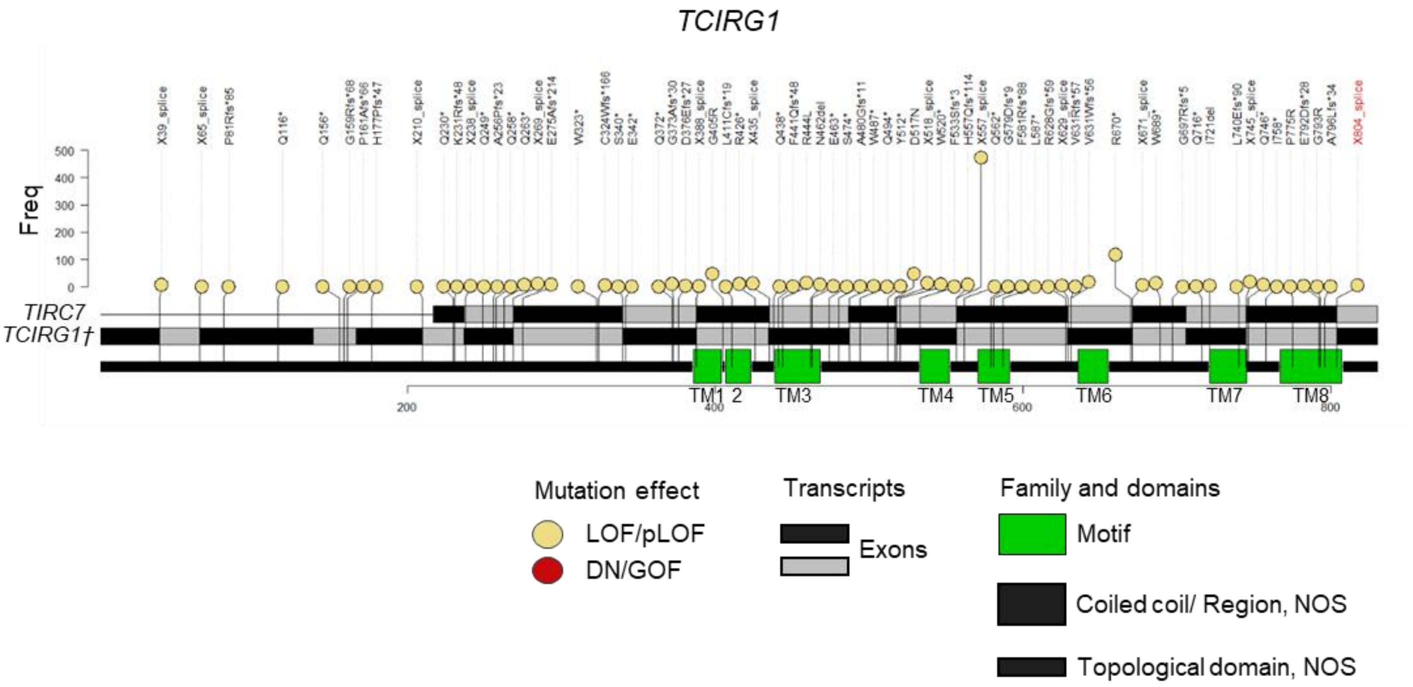
TCIRG1
X39_splice
X65_splice
P81Rfs*85
Q116*
Q156*
G159Rfs*68
P161Afs*66
H177Pfs*47
X210_splice
Q230*
K231Rfs*48
X238_splice
Q249*
A256Pfs*23
Q258*
Q263*
X269_splice
E275Afs*214
W323*
C324Wfs*166
S340*
E342*
Q372*
G373Afs*30
D376Efs*27
X388_splice
G405R
L411Cfs*19
R426*
X435_splice
Q438*
F441Qfs*48
R444L
N462del
E463*
S474*
A480Gfs*11
W487*
Q494*
Y512*
D517N
X518_splice
W520*
F533Sfs*3
H557Qfs*114
X557_splice
Q562*
G579Dfs*9
F581Rfs*88
L587*
R628Gfs*59
X629_splice
V631Rfs*57
V631Wfs*56
R670*
X671_splice
W689*
G697Rfs*5
Q716*
I721del
L740Efs*90
X745_splice
Q746*
I758*
P775R
E792Dfs*28
G793R
A796Lfs*34
X804_splice
Freq
500
400
300
200
100
0
TIRC7
TCIRG1†
TM1
2
TM3
TM4
TM5
TM6
TM7
TM8
200
400
600
800
Mutation effect
LOF/pLOF
DN/GOF
Transcripts
Exons
Family and domains
Motif
Coiled coil/ Region, NOS
Topological domain, NOS

Supplemental Figure 4. Clinical, laboratory and genetic characterization of patients with high-confidence deleterious (hcD) variants associated with dominant IEI. A) Clinical and laboratory features of the carriers of hcD variants (hcD, red) vs. non-carriers (NC, gray). *: $p<0.05$, **: $p<0.01$, ***: $p<0.001$. Exact p values are shown for marginally significant p-values ($0.05 < p < 0.10$). Abbreviations: ALC: absolute lymphocyte count. ANC: absolute neutrophil count. WBC: white blood cells. Hb: Hemoglobin. LGLs: large granular lymphocyte count.

Suppl. Fig.4

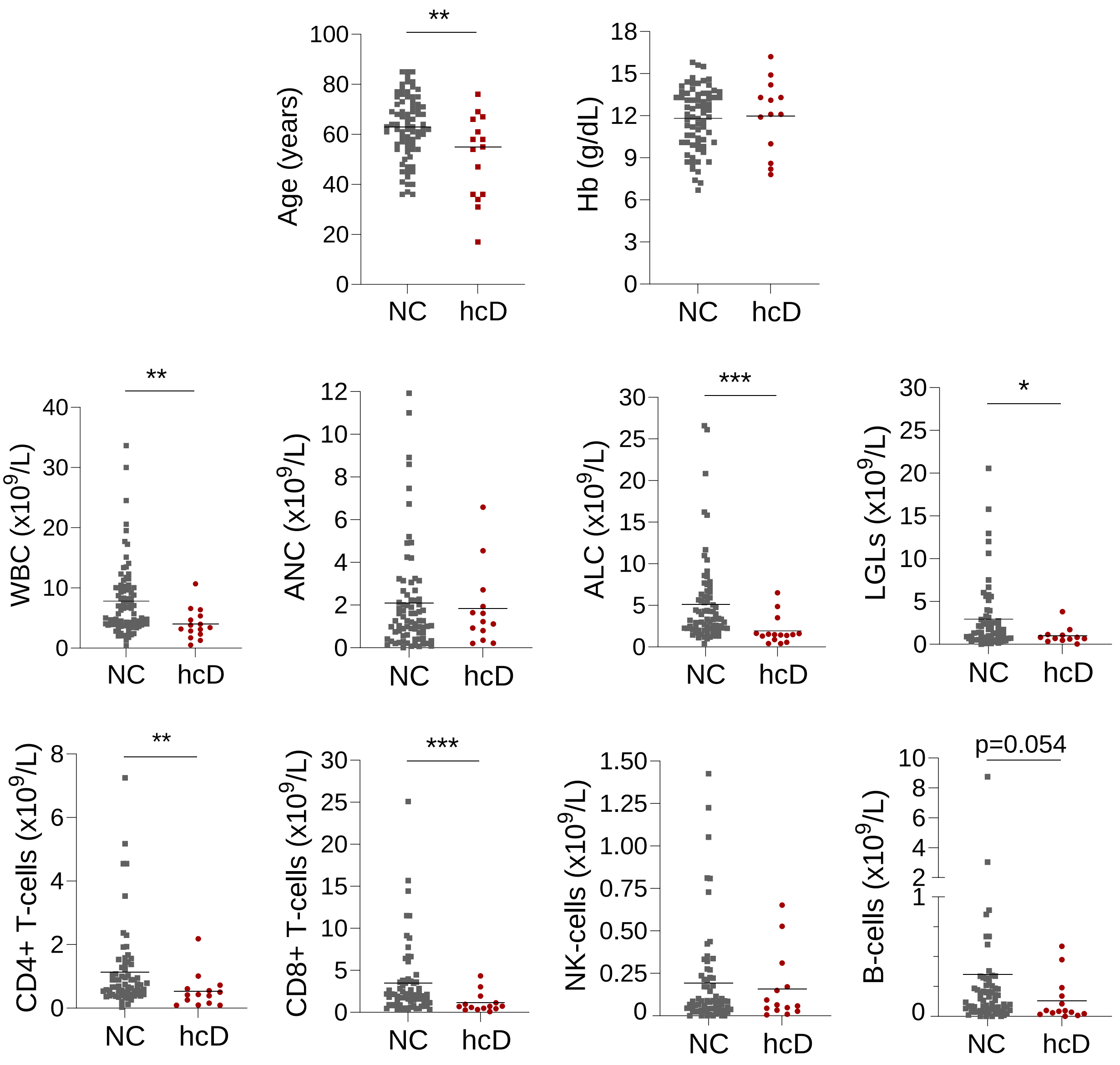

Age (years)
**
NC
hcD
Hb (g/dL)
WBC (x10^9/L)
ANC (x10^9/L)
ALC (x10^9/L)
***
LGLs (x10^9/L)
*
CD4+ T-cells (x10^9/L)
CD8+ T-cells (x10^9/L)
NK-cells (x10^9/L)
B-cells (x10^9/L)
p=0.054

Supplemental Figure 5. Survival outcomes for carriers of high-confidence deleterious variants (hcD). A) Kaplan Meier curves showing the EFS stratified by the presence of hcD variants. Log-rank P-value=0.18. B) Kaplan Meier curves showing the OS stratified by the presence of hcD variants. Log-rank P-value=0.11. EFS: event-free survival. OS: overall survival.

Suppl. Fig.5

A

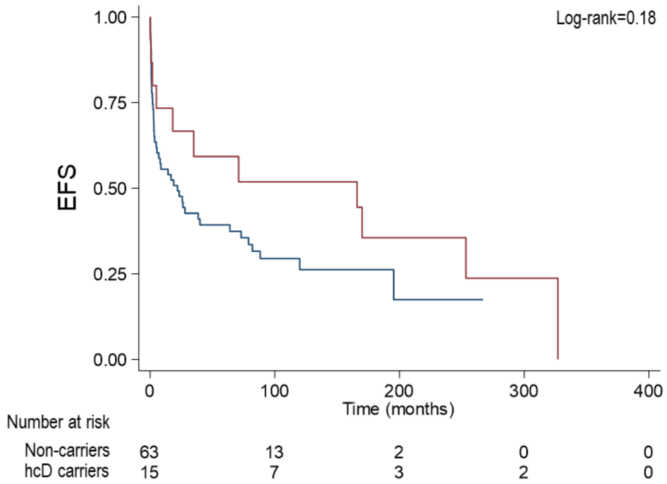

B

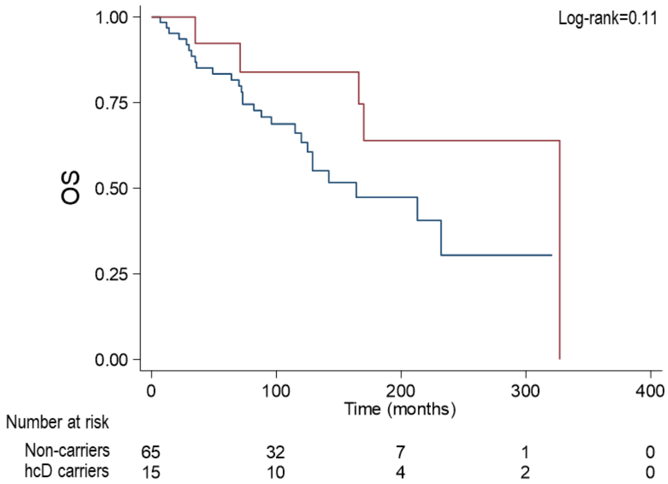

Supplemental Figure 6. CNLog2 of *STAT3*-related genes and TP53 inferred from our WES cohort. The reference lines represent the thresholds to define CN gains (upper line, red) and CN losses (lower line, blue). Each dot represents a patient sample.

Suppl. Fig.6

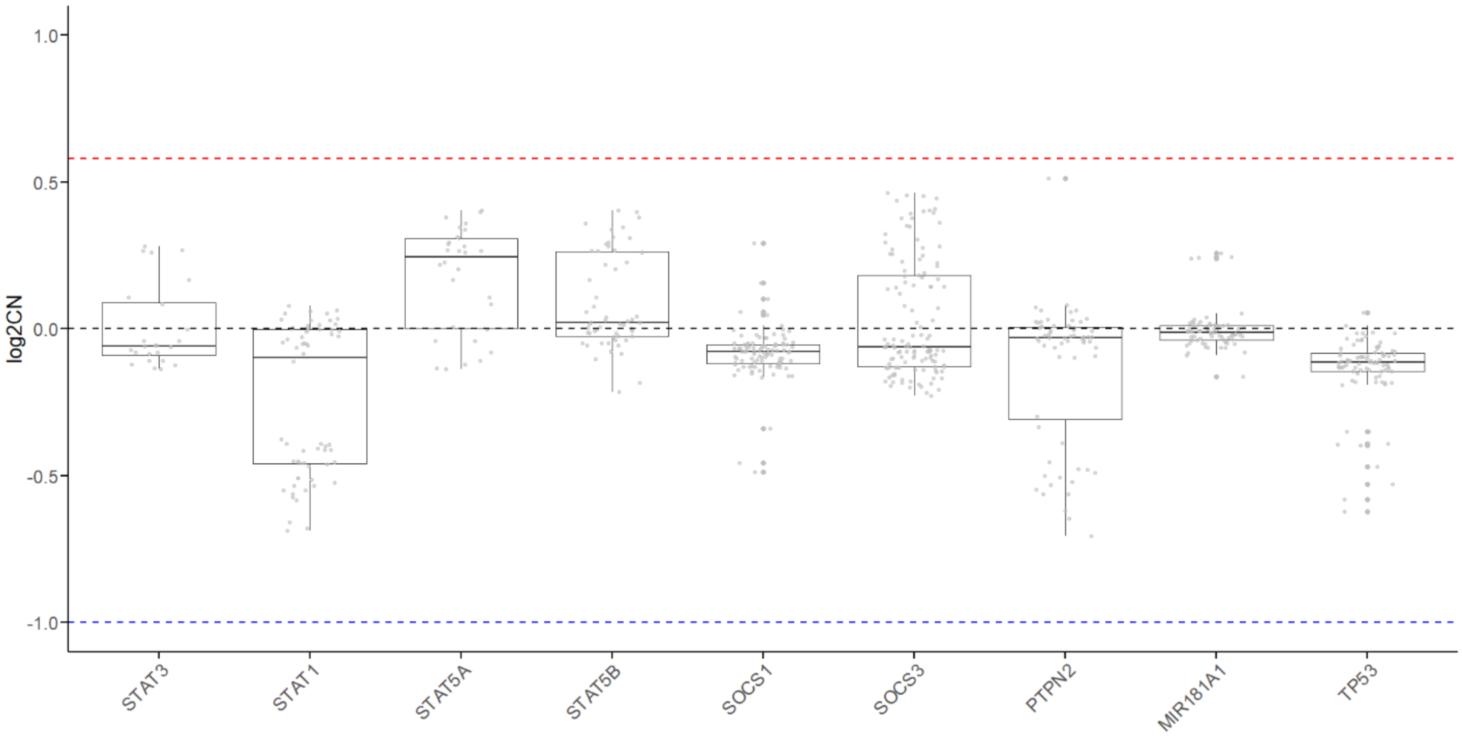

log2CN
1.0
0.5
0.0
-0.5
-1.0
STAT3
STAT1
STAT5A
STAT5B
SOCS1
SOCS3
PTPN2
MIR181A1
TP53

Supplemental Figure 7. CNLog2 of *STAT3*-related genes and TP53 from 26 Mature T NK Neoplasms as part of the DepMap Data Release. The reference lines represent the thresholds to define CN gains (upper line, red) and CN losses (lower line, blue). Cell lines with CNVs are labeled. The T-LGLL cell line MOTN1 is labelled in all columns regardless the gene CN status.

Suppl. Fig.7

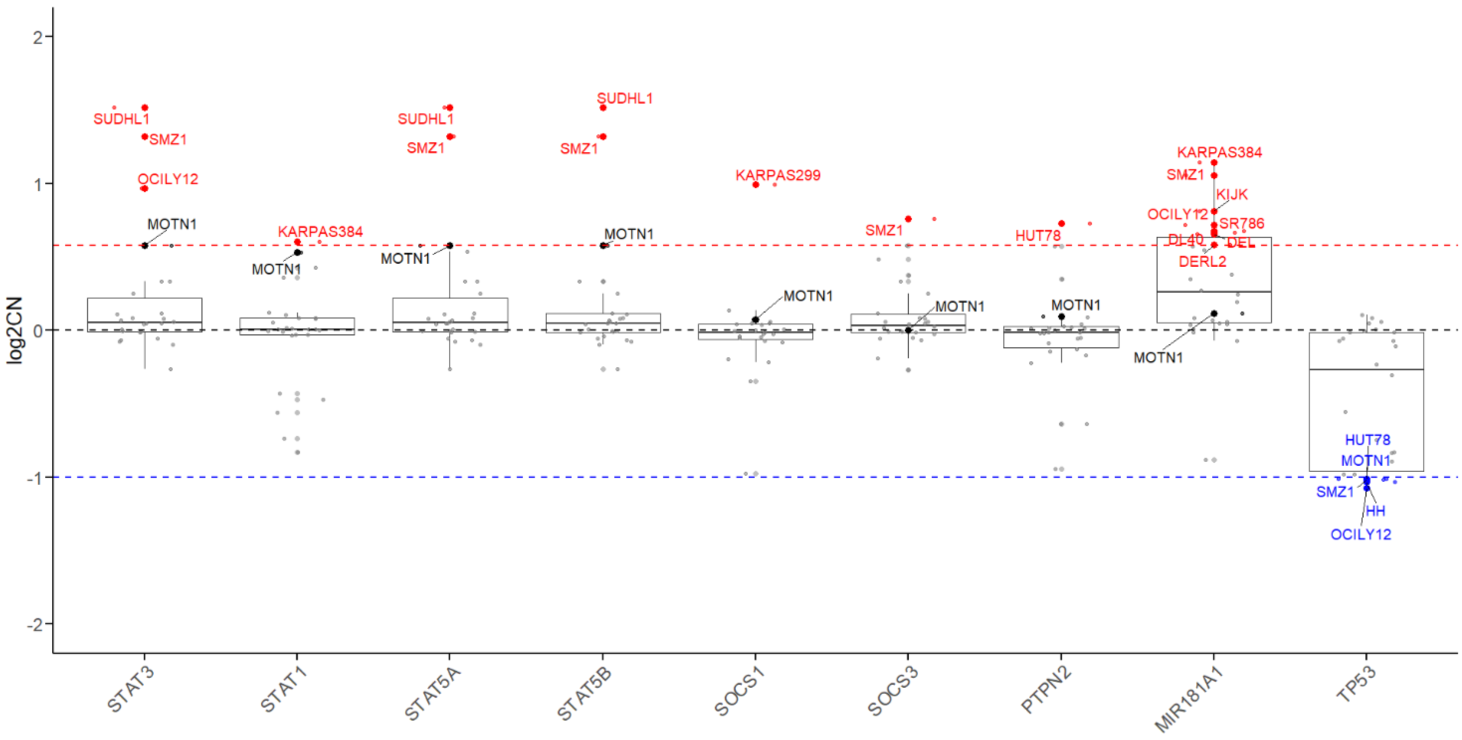

log2CN
2
1
0
-1
-2
SUDHL1
SMZ1
OCILY12
MOTN1
KARPAS384
MOTN1
SUDHL1
SMZ1
MOTN1
SUDHL1
SMZ1
MOTN1
KARPAS299
MOTN1
SMZ1
MOTN1
HUT78
MOTN1
KARPAS384
SMZ1
KIJK
OCILY12
SR786
DL40
DEL
DERL2
MOTN1
HUT78
MOTN1
SMZ1
HH
OCILY12
STAT3
STAT1
STAT5A
STAT5B
SOCS1
SOCS3
PTPN2
MIR181A1
TP53

Supplemental Figure 8. Mutational plot with coupled damaging SNV and CNV data from 26 mature T neoplasms as part of the DepMap Data Release. The plot represents the mutational status of top mutated T-cell lymphoid drivers assessed in our WES patient cohort. Heatmap clustering was performed with Ward's linkage. Values are scaled for each column. Amp: amplification. SNV: single nucleotide variant. CNV: copy number variant. ALCL: Anaplastic large cell lymphoma. CTCL: cutaneous T cell lymphoma. ATLL: adult T-cell leukemia/lymphoma. HSTCL: hepatosplenic T-cell lymphoma. T-LGLL: T-cell large granular lymphocytic leukemia. PTCL: peripheral T cell lymphoma. Ploidy is abbreviated as 2n: near-diploid, 2n+: hyperdiploid, 3n: triploid, 4n: tetraploid.

Suppl. Fig.8

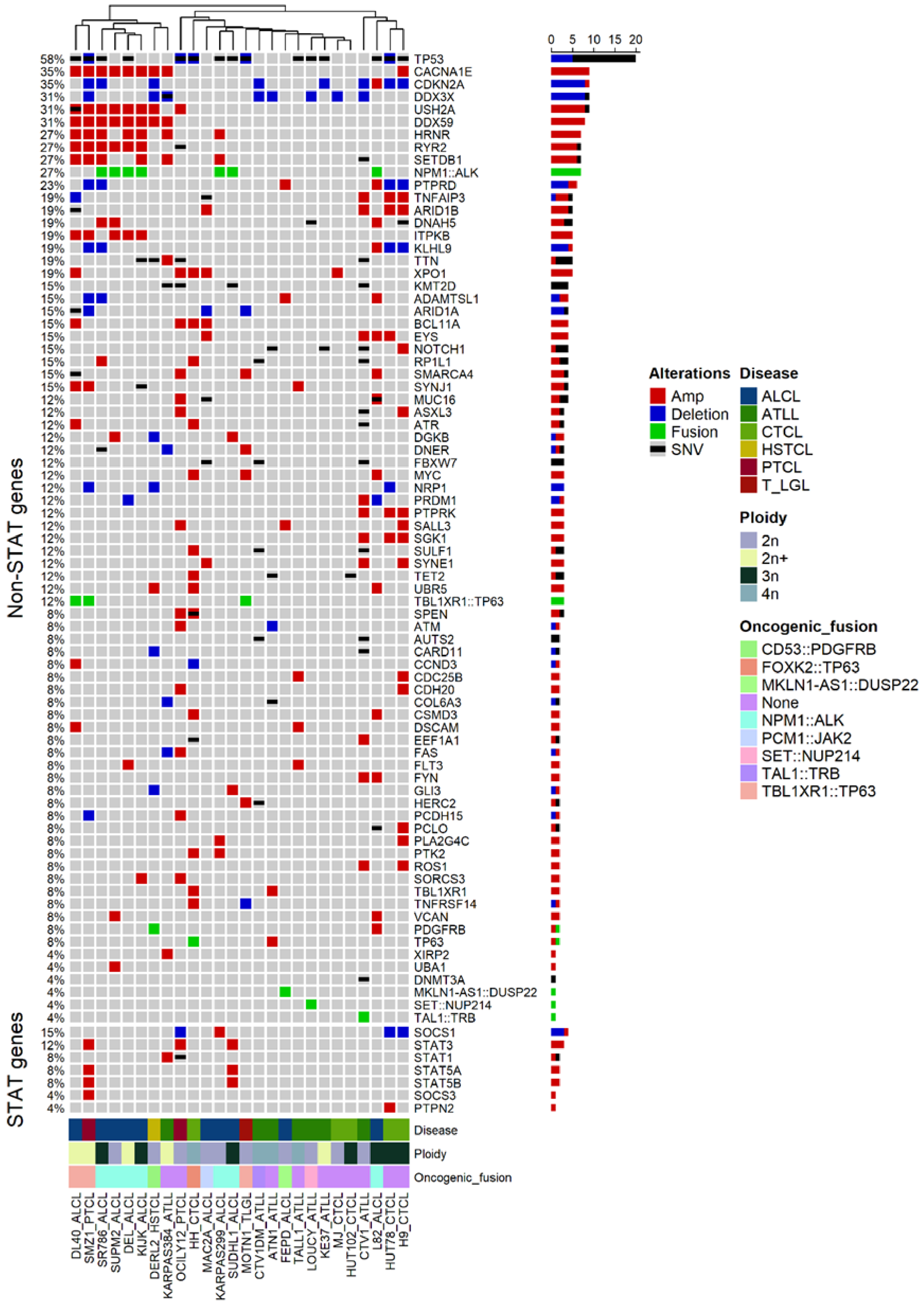
Non-STAT genes
STAT genes
0 5 10 15 20
58% TP53
35% CACNA1E
35% CDKN2A
31% DDX3X
31% USH2A
31% DDX59
27% HRNR
27% RYR2
27% SETDB1
27% NPM1::ALK
23% PTPRD
19% TNFAIP3
19% ARID1B
19% DNAH5
19% ITPKB
19% KLHL9
19% TTN
19% XPO1
15% KMT2D
15% ADAMTSL1
15% ARID1A
15% BCL11A
15% EYS
15% NOTCH1
15% RP1L1
15% SMARCA4
15% SYNJ1
12% MUC16
12% ASXL3
12% ATR
12% DGKB
12% DNER
12% FBXW7
12% MYC
12% NRP1
12% PRDM1
12% PTPRK
12% SALL3
12% SGK1
12% SULF1
12% SYNE1
12% TET2
12% UBR5
12% TBL1XR1::TP63
8% SPEN
8% ATM
8% AUTS2
8% CARD11
8% CCND3
8% CDC25B
8% CDH20
8% COL6A3
8% CSMD3
8% DSCAM
8% EEF1A1
8% FAS
8% FLT3
8% FYN
8% GLI3
8% HERC2
8% PCDH15
8% PCLO
8% PLA2G4C
8% PTK2
8% ROS1
8% SORCS3
8% TBL1XR1
8% TNFRSF14
8% VCAN
8% PDGFRB
8% TP63
4% XIRP2
4% UBA1
4% DNMT3A
4% MKLN1-AS1::DUSP22
4% SET::NUP214
4% TAL1::TRB
15% SOCS1
12% STAT3
8% STAT1
8% STAT5A
8% STAT5B
4% SOCS3
4% PTPN2
Disease
Ploidy
Oncogenic_fusion
DL40_ALCL
SMZ1_PTCL
SR786_ALCL
SUPM2_ALCL
DEL_ALCL
KIJK_ALCL
DERL2_HSTCL
KARPAS384_ATLL
OCILY12_PTCL
HH_CTCL
MAC2A_ALCL
KARPAS299_ALCL
SUDHL1_ALCL
MOTN1_TLGL
CTV1DM_ATLL
ATN1_ATLL
FEPD_ALCL
TALL1_ATLL
LOUCY_ATLL
KE37_ATLL
MJ_CTCL
HUT102_CTCL
CTV1_ATLL
L82_ALCL
HUT78_CTCL
H9_CTCL
Alterations
Amp
Deletion
Fusion
SNV
Disease
ALCL
ATLL
CTCL
HSTCL
PTCL
T_LGL
Ploidy
2n
2n+
3n
4n
Oncogenic_fusion
CD53::PDGFRB
FOXK2::TP63
MKLN1-AS1::DUSP22
None
NPM1::ALK
PCM1::JAK2
SET::NUP214
TAL1::TRB
TBL1XR1::TP63

Supplemental Figure 9. **Co-mutation analysis of T-cell lymphoid drivers in patients with T-LGLL according to the IEI hcD variant status.** A) Gene pathway analysis of the genes with hcD variants (red) and T-LGLL dysregulated pathways (gray) using GeneMANIA and Cytoscape. The Venn diagram summarizes the number of gene pathways enriched and shared by the networks, which are further detailed in Supplementary Table 7. A) Correlation plot represents positive and negative associations of mutations in T-cell lymphoid drivers and IEI hcD variants, grouped at the bottom of the plot as immune dysregulation (hyperactive responses) and immunodeficiency (defective responses). Only correlations with p-values $p<0.10$ are shown. B) Enrichment analysis of the HALLMARK pathways involved by the T-cell lymphoid drivers associated with patients with IEI hcD variants. Top 5 pathways enriched (q-value < 0.10) in patients with immune dysregulation (normalized enrichment score [NES] > 0, right) and immunodeficient (NES < 0) hcD variants are shown.

Suppl. Fig.9

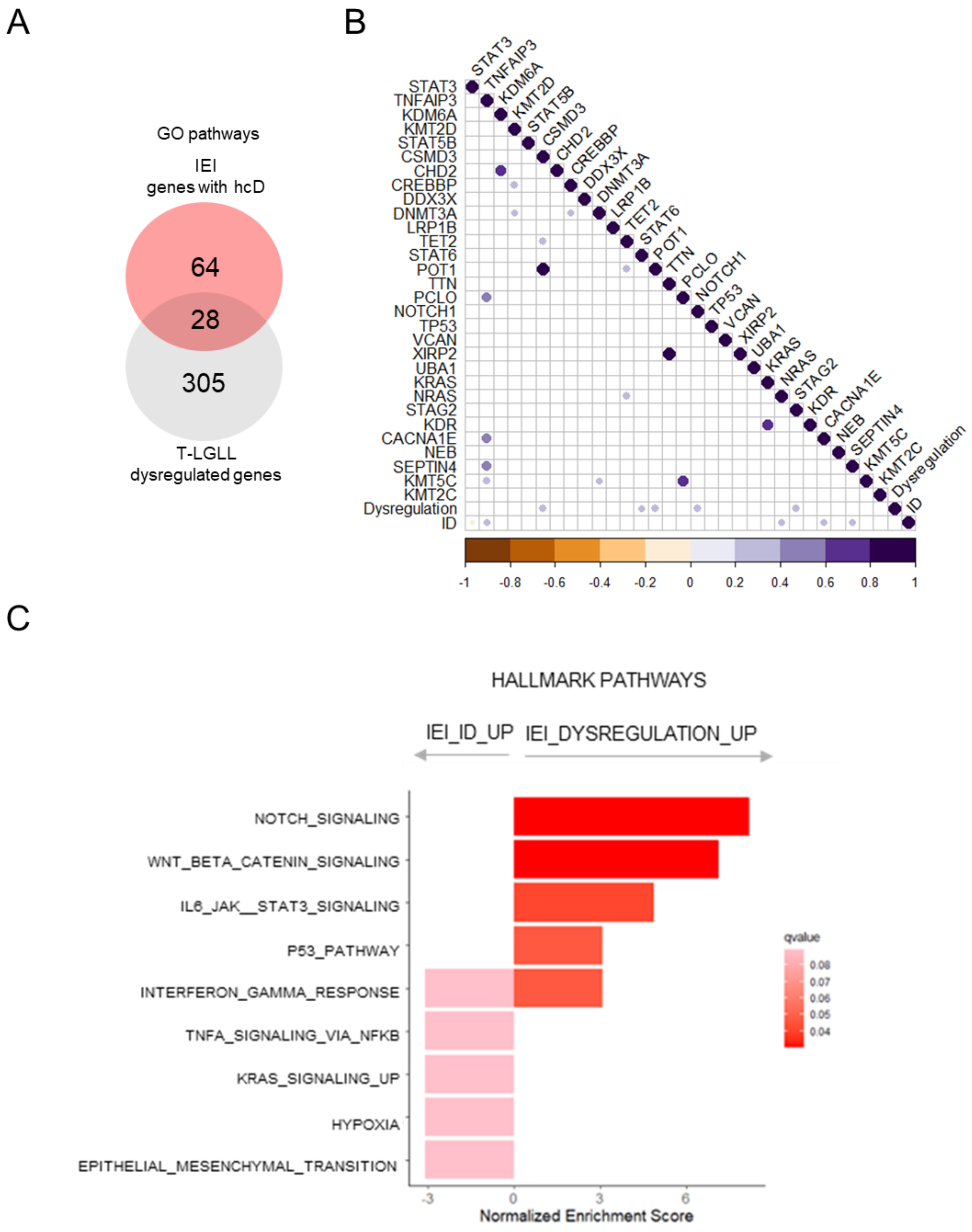

A
GO pathways
IEI
genes with hcD
64
28
305
T-LGLL
dysregulated genes
B
STAT3
TNFAIP3
KDM6A
KMT2D
STAT5B
CSMD3
CHD2
CREBBP
DDX3X
DNMT3A
LRP1B
TET2
STAT6
POT1
TTN
PCLO
NOTCH1
TP53
VCAN
XIRP2
UBA1
KRAS
NRAS
STAG2
KDR
CACNA1E
NEB
SEPTIN4
KMT5C
KMT2C
Dysregulation
ID
-1
-0.8
-0.6
-0.4
-0.2
0
0.2
0.4
0.6
0.8
1
C
HALLMARK PATHWAYS
IEI_ID_UP
IEI_DYSREGULATION_UP
NOTCH_SIGNALING
WNT_BETA_CATENIN_SIGNALING
IL6_JAK__STAT3_SIGNALING
P53_PATHWAY
INTERFERON_GAMMA_RESPONSE
TNFA_SIGNALING_VIA_NFKB
KRAS_SIGNALING_UP
HYPOXIA
EPITHELIAL_MESENCHYMAL_TRANSITION
-3
0
3
6
Normalized Enrichment Score
qvalue
0.08
0.07
0.06
0.05
0.04

Supplemental Figure 10. Single-cell RNA+TCRαβ-seq in T-LGLL and healthy control samples. A) Focused UMAP of the hyperexpanded T cells (>10 templates) and inferred *STAT3*mt status from T-LGLL and healthy control samples. The admixed barplot represents the proportion of the cells according to the *STAT3*mt status per sample. B) Focused UMAP of the hyperexpanded T-cells and inferred T-cells subtypes from T-LGLL and healthy control samples. The admixed barplot represents the proportion of the cells according to the *STAT3*mt status per sample. C) Fraction of cells (%) expressing common T-cell markers. TCM: T central memory cells. TEM: T effector memory cells. CLP: common lymphoid progenitor. Tregs: T regulatory cells. The category “rare” aggregates infrequent CD8+ T cell populations.

Suppl. Fig. 10.

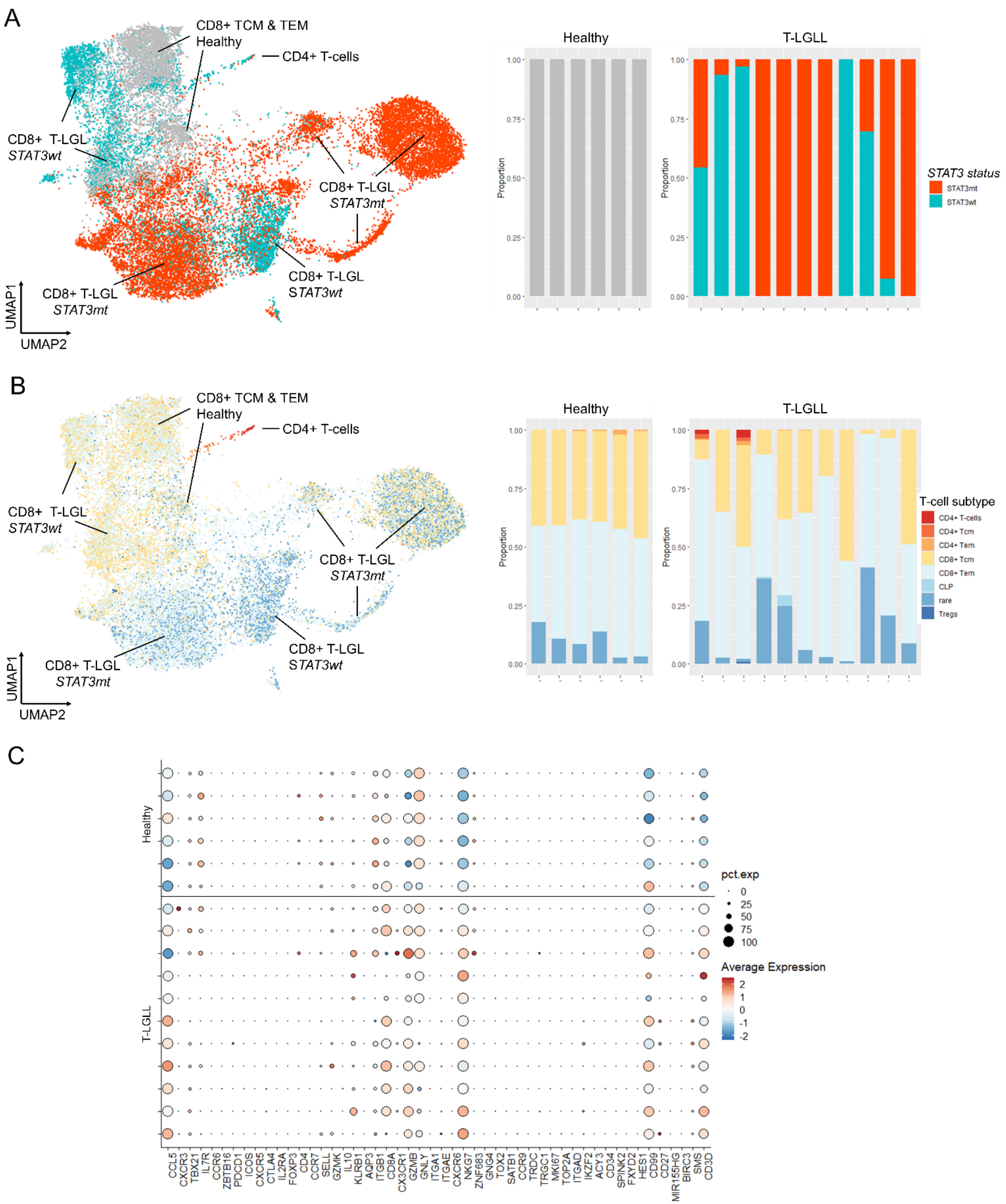
A
CD8+ TCM & TEM
Healthy
CD4+ T-cells
CD8+ T-LGL
STAT3wt
CD8+ T-LGL
STAT3mt
CD8+ T-LGL
STAT3wt
CD8+ T-LGL
STAT3mt
UMAP1
UMAP2
Healthy
T-LGLL
Proportion
1.00
0.75
0.50
0.25
0.00
STAT3 status
STAT3mt
STAT3wt
B
CD8+ TCM & TEM
Healthy
CD4+ T-cells
CD8+ T-LGL
STAT3wt
CD8+ T-LGL
STAT3mt
CD8+ T-LGL
STAT3wt
CD8+ T-LGL
STAT3mt
UMAP1
UMAP2
Healthy
T-LGLL
Proportion
T-cell subtype
CD4+ T-cells
CD4+ Tcm
CD4+ Tem
CD8+ Tcm
CD8+ Tem
CLP
rare
Tregs
C
Healthy
T-LGLL
pct.exp
0
25
50
75
100
Average Expression
2
1
0
-1
-2
CCL5 CXCR3 TBX21 IL7R CCR6 ZBTB16 PDCD1 ICOS CXCR5 CTLA4 IL2RA FOXP3 CD4 CCR7 SELL GZMK IL10 KLRB1 AQP3 ITGB1 CD8A CX3CR1 GZMB GNLY ITGA1 ITGAE CXCR6 NKG7 ZNF683 GNG4 TOX2 SATB1 CCR9 TRDC TRGC1 MKI67 TOP2A ITGAD IKZF2 ACY3 CD34 SPINK2 FXYD2 HES1 CD99 CD27 MIR155HG BIRC3 SMS CD3D

Supplemental Figure 11. Single-cell RNA+TCRαβ-seq analysis of *STAT3*-related genes in T-LGLL and healthy control samples. A) Fraction of cells (%) expressing *STAT3* and *STAT3*-related positive/negative regulators. B) Scaled expression of *STAT3*-related genes between T-LGLL clusters highlighted in the same UMAP representation (upper panels) and expression levels between *STAT3*mt vs. *STAT3* wt T-LGLL and healthy controls (t-test p-values).

Suppl. Fig. 11.

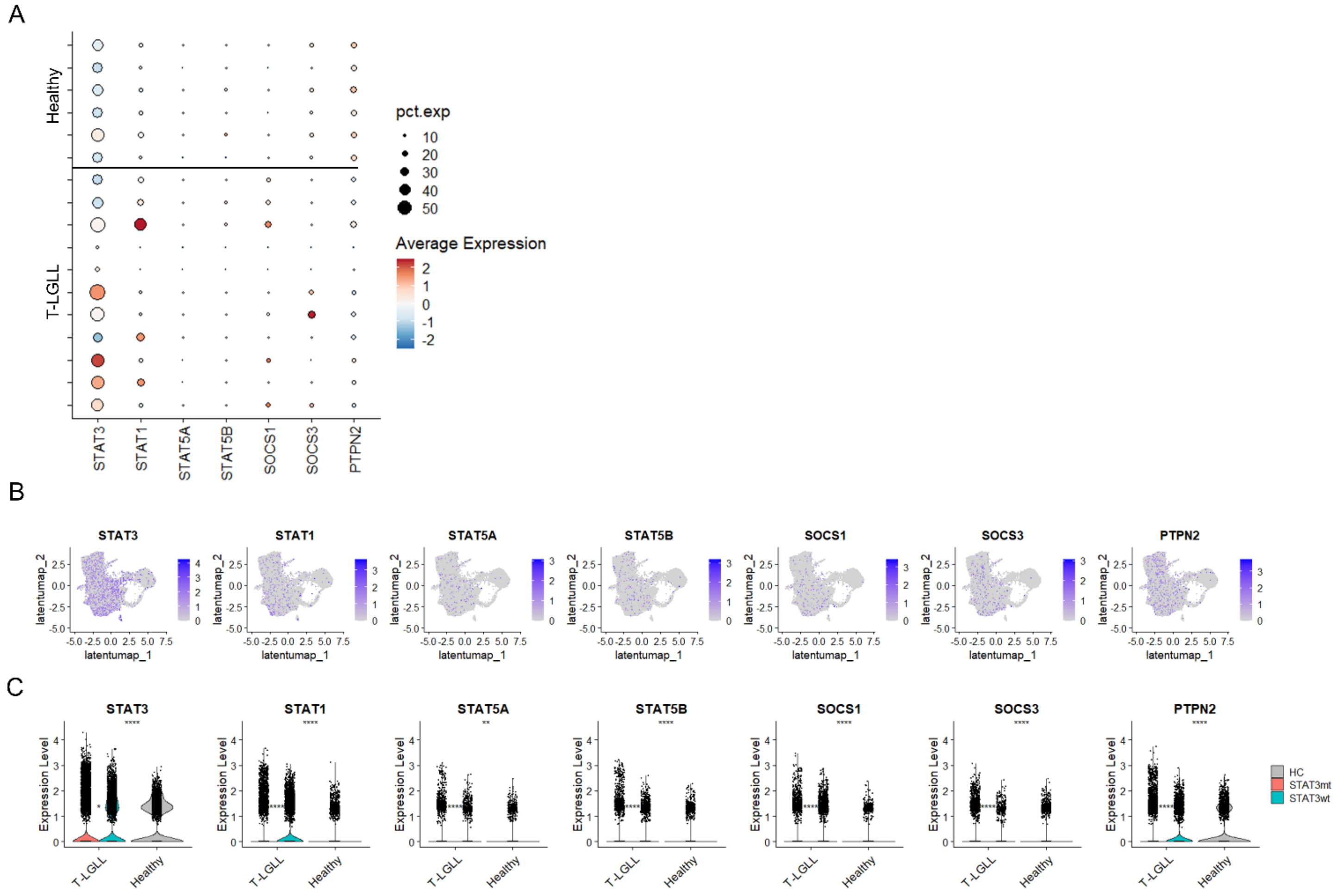
A
Healthy
T-LGLL
pct.exp
10
20
30
40
50
Average Expression
2
1
0
-1
-2
STAT3
STAT1
STAT5A
STAT5B
SOCS1
SOCS3
PTPN2
B
STAT3
STAT1
STAT5A
STAT5B
SOCS1
SOCS3
PTPN2
latentumap_2
latentumap_1
C
STAT3
STAT1
STAT5A
STAT5B
SOCS1
SOCS3
PTPN2
Expression Level
T-LGLL
Healthy
HC
STAT3mt
STAT3wt

Supplemental Figure 12. Single-cell RNA+TCRαβ-seq analysis of *STAT3*-related genes in T-LGLL and healthy control samples. A) Fraction of the cells expressing TCR-related genes. B) Scaled expression of TCR-related genes between groups highlighted in the same UMAP representation. C) Expression levels between *STAT3*mt vs. *STAT3* wt T-LGLL and healthy controls (t-test P-values). *: p<0.05, **: p<0.01, ***: p<0.001, ****: p<0.0001.

Suppl. Fig.12

A

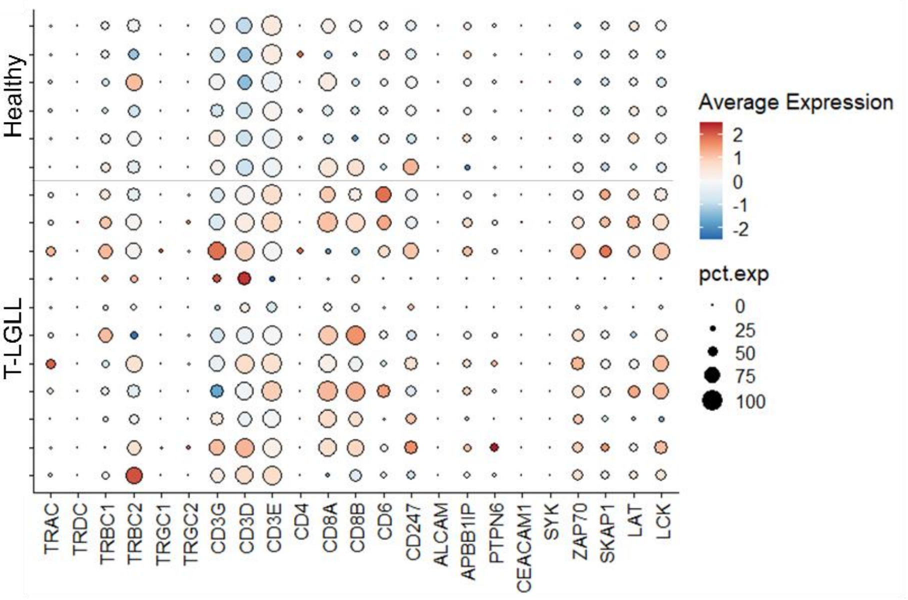

B

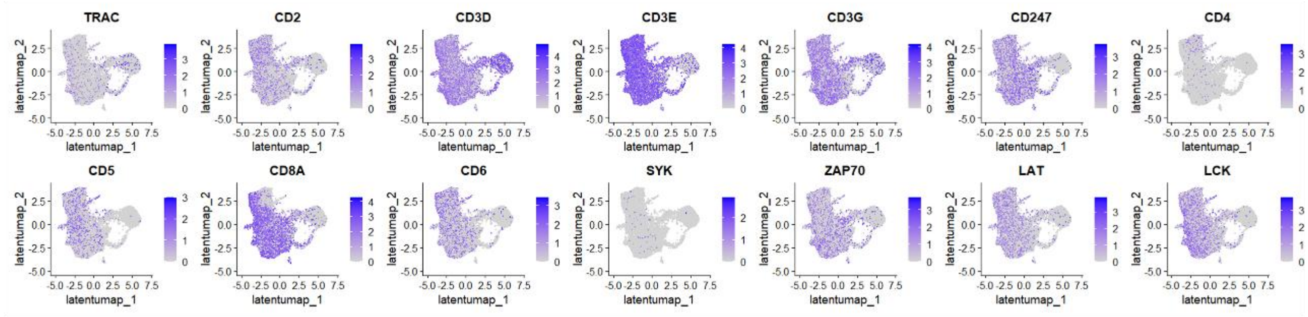

C

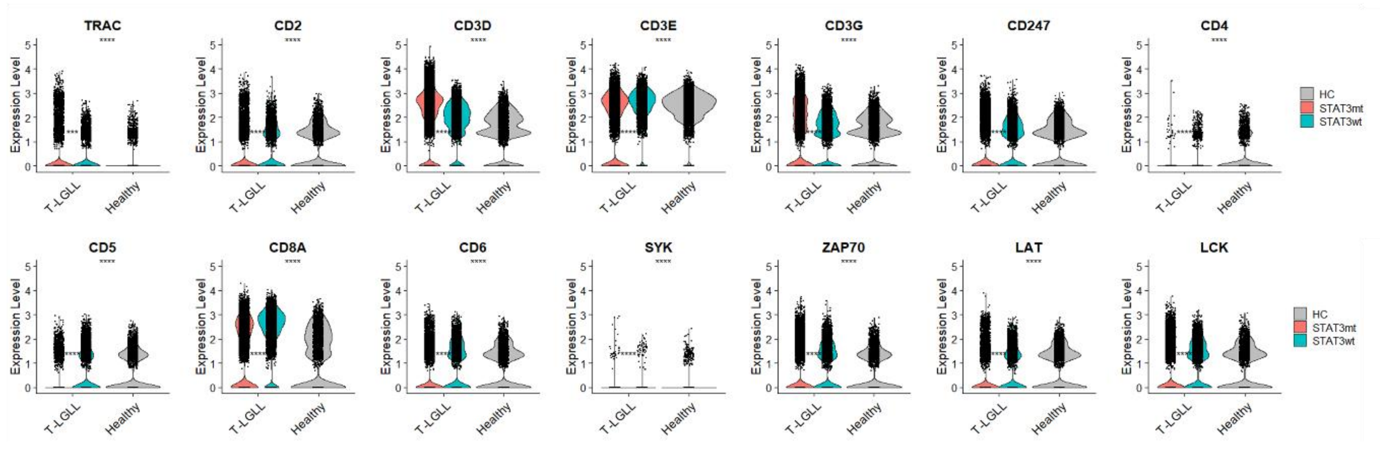

Supplemental Figure 13. Bulk-RNA-seq analysis of *STAT3*- and TCR-related genes in mature T-cell lymphoma/leukemia cell lines from DepMap. A) Normalized mRNA expression levels of *STAT3* and related genes in T-cell lymphoma/leukemia cell lines according to the *STAT3* amplification status (Wilcoxon test P-values). Normalized mRNA expression levels of TCR-related genes in T-cell lymphoma/leukemia cell lines according to the *STAT3* amplification status (Wilcoxon test P-values). AMP: *STAT3* amplification. WT: *STAT3* neutral CN. *: p<0.05, **: p<0.01, ***: p<0.001, ****: p<0.0001. Exact p-values marginally significant (0.05 < p < 0.10) are shown.

Suppl. Fig.13

A

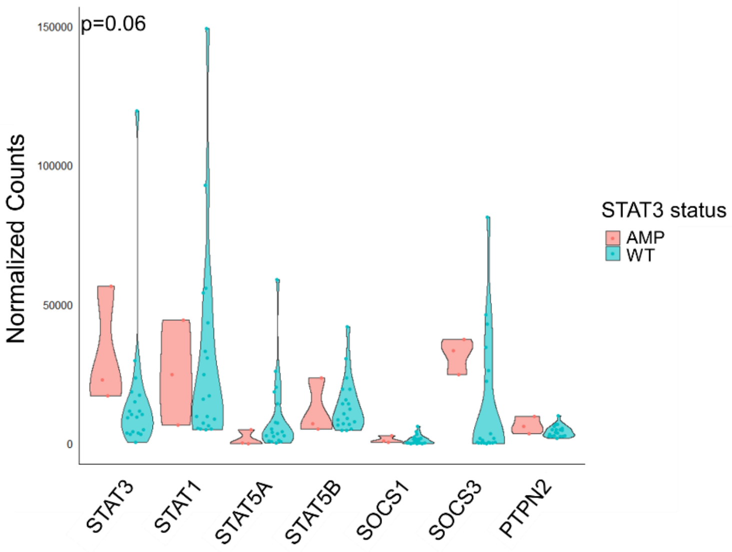

B

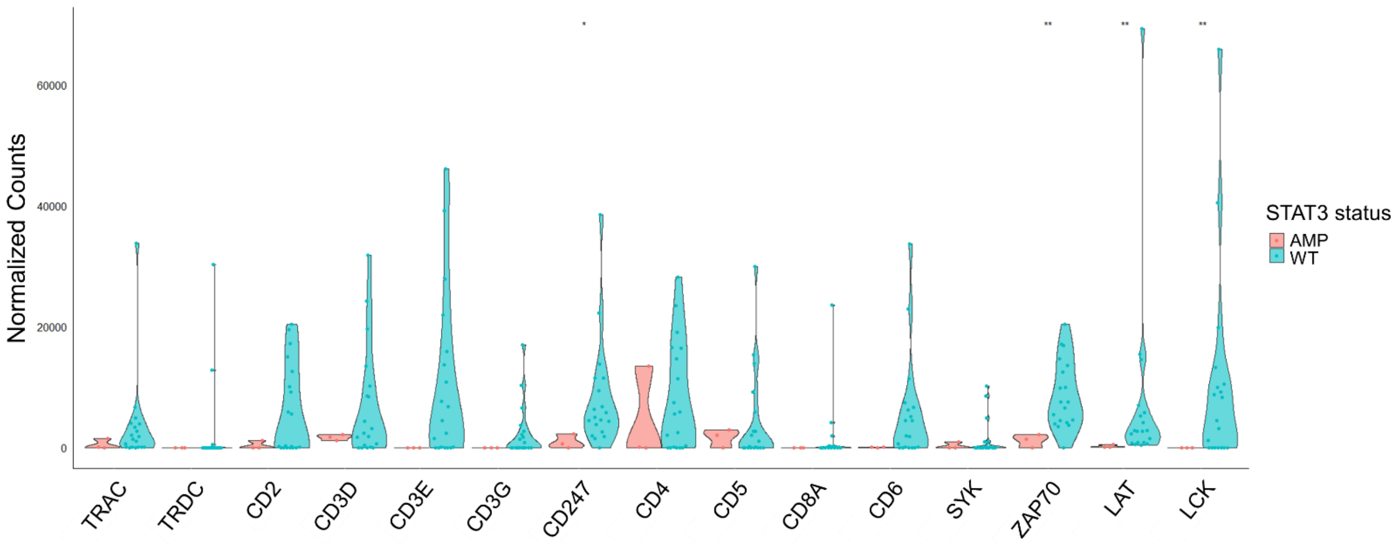

Supplemental Figure 14. Bulk-RNA-seq differential gene expression analysis between *STAT3*amp and *STAT3*wt T-cell lymphoma/leukemia lines from DepMap. The *STAT3*amp and fusion-matched-*STAT3*wt pairs of cell lines were defined based on the presence/absence of a specific oncogene fusion in the *STAT3*amp line, as follows: A) Set1: TP63-rearranged ; B) Set2: ALK-rearranged; C) Set3) No gene fusion. The upper plots represent differentially expressed genes (Bonferroni corrected padj < 0.05 two-sided Wilcoxon test). Top 50 genes are labeled. The x-axis denotes the average log2 fold-change between the two conditions and y-axis the padj in a -log10 transformed scale. The lower plots show top upregulated and downregulated GO-pathways (q-value < 0.15, padj < 0.05, Benjamini-Hochberg corrected Fisher's exact test on differentially expressed genes) in *STAT3*amp vs. *STAT3*wt lines. AMP: *STAT3* amplification. WT: *STAT3* neutral CN.

Suppl. Fig.14

A

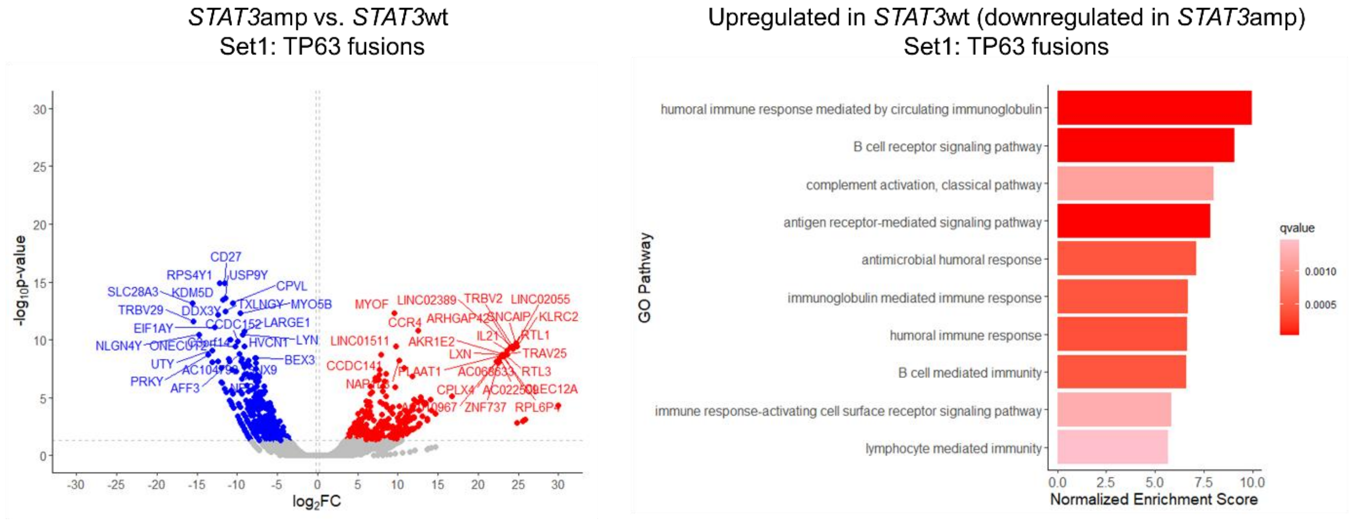

B

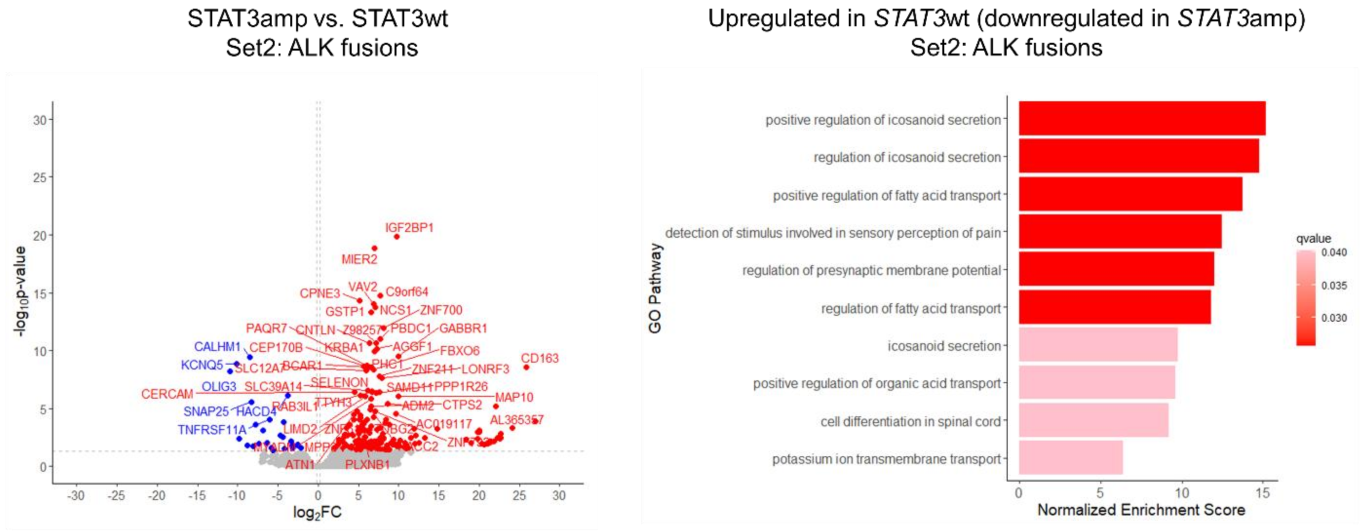

C

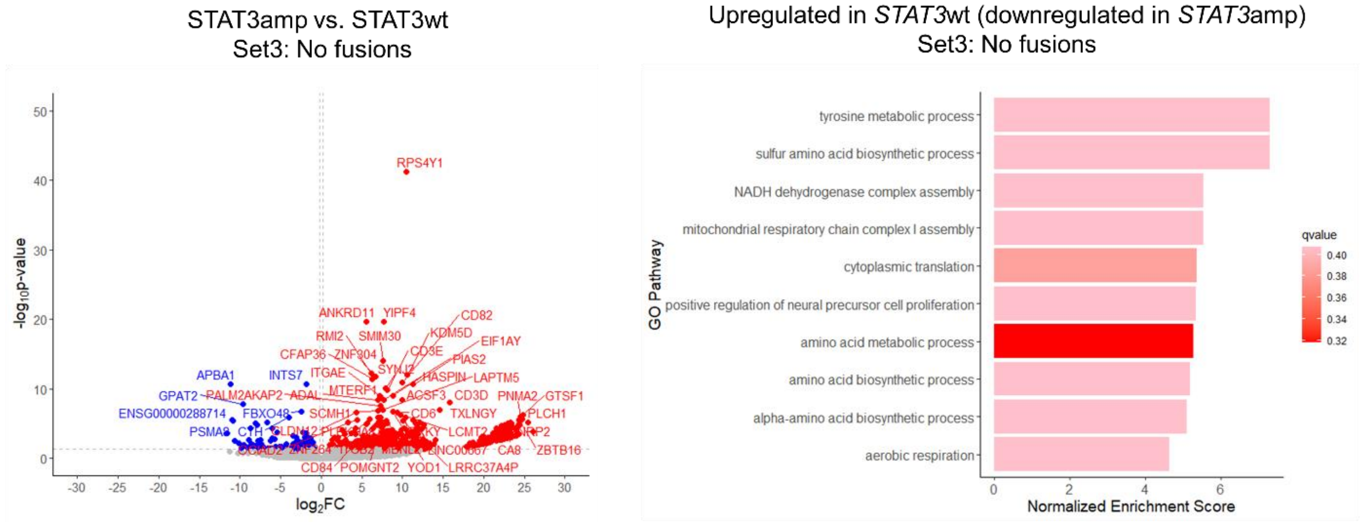

Supplemental Figure 15. TCR signalosome score in *STAT3*mt vs. *STAT3*wt cells. A) scRNA-seq from T-LGLL and healthy control samples. The violin and ridge plots show the TCR score in *STAT3*mt, *STAT3*wt T-LGL clones and in HC hyperexpanded T-cells. B) Bulk RNA seq from T-cell lymphoma/leukemia cell lines from DepMap. The violin and ridge plots show the TCR score in *STAT3*amp vs. *STAT3*wt cell lines. Wilcoxon test p<0.10 are shown. *: p <0.05, **: p<0.01, ***: p<0.001, ****: p < 0.0001. Exact p-values marginally significant (0.05 < p < 0.10) are shown.

Suppl. Fig.15

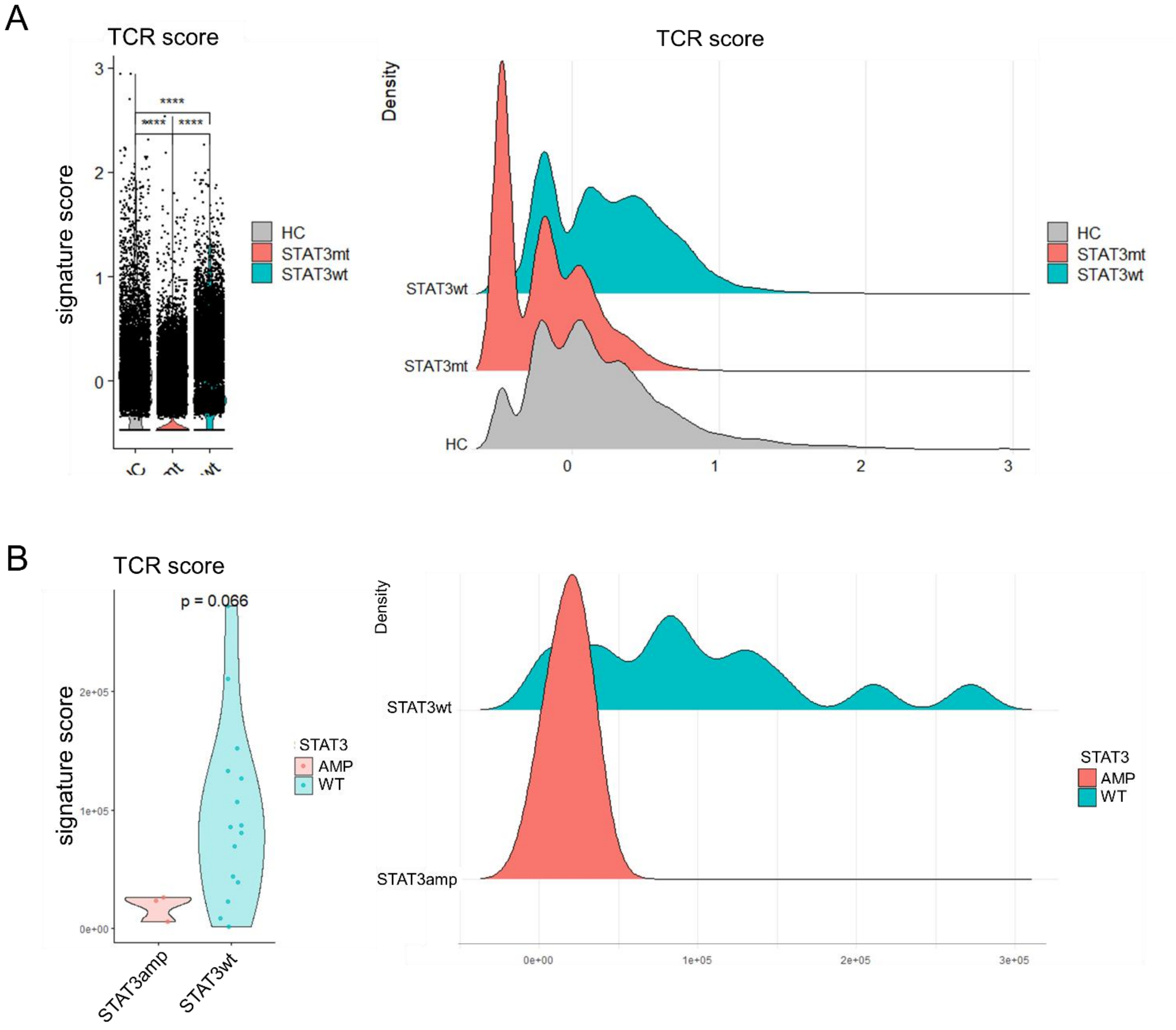
A
TCR score
signature score
HC
STAT3mt
STAT3wt
TCR score
Density
STAT3wt
STAT3mt
HC
HC
STAT3mt
STAT3wt
0
1
2
3
B
TCR score
p = 0.066
signature score
STAT3
AMP
WT
STAT3amp
STAT3wt
Density
STAT3wt
STAT3amp
STAT3
AMP
WT
0e+00
1e+05
2e+05
3e+05

References

1. Sanikommu SR, et al. Clinical features and treatment outcomes in large granular lymphocytic leukemia (LGLL). *Leuk Lymphoma*. 2018;59(2):416–422.

2. Zawit M, et al. Large Granular Lymphocytic Leukemia: From Immunopathogenesis to Treatment of Refractory Disease. *Cancers (Basel)*. 2021;13(17). https://doi.org/10.3390/cancers13174418.

3. Alaggio R, et al. The 5th edition of the World Health Organization Classification of Haematolymphoid Tumours: Lymphoid Neoplasms. *Leukemia*. 2022;36(7):1720–1748.

4. Compagno N, et al. Immunoglobulin replacement therapy in secondary hypogammaglobulinemia. *Front Immunol*. 2014;5:626.

5. Andersson E, et al. Activating somatic mutations outside the SH2-domain of STAT3 in LGL leukemia. *Leukemia*. 2016;30(5):1204–1208.

6. Gurnari C, et al. A study of Telomerase Reverse Transcriptase rare variants in myeloid neoplasia. *Hematol Oncol*. 2022;40(4):812–817.

7. Adema V, et al. Pathophysiologic and clinical implications of molecular profiles resultant from deletion 5q. *EBioMedicine*. 2022;80:104059.

8. Li H, Durbin R. Fast and accurate long-read alignment with Burrows-Wheeler transform. *Bioinformatics*. 2010;26(5):589–595.

9. McKenna A, et al. The Genome Analysis Toolkit: a MapReduce framework for analyzing next-generation DNA sequencing data. *Genome Res*. 2010;20(9):1297–1303.

10. Wang K, Li M, Hakonarson H. ANNOVAR: functional annotation of genetic variants from high-throughput sequencing data. *Nucleic Acids Res*. 2010;38(16):e164.

11. Bousfiha A, et al. The 2022 Update of IUIS Phenotypical Classification for Human Inborn Errors of Immunity. *J Clin Immunol*. 2022;42(7):1508–1520.

12. Landrum MJ, et al. ClinVar: improving access to variant interpretations and supporting evidence. *Nucleic Acids Res*. 2018;46(D1):D1062–D1067.

13. Kopanos C, et al. VarSome: the human genomic variant search engine. *Bioinformatics*. 2019;35(11):1978–1980.

14. Ng PC, Henikoff S. SIFT: Predicting amino acid changes that affect protein function. *Nucleic Acids Res*. 2003;31(13):3812–3814.

15. Choi Y, Chan AP. PROVEAN web server: a tool to predict the functional effect of amino acid substitutions and indels. *Bioinformatics*. 2015;31(16):2745–2747.

16. Chun S, Fay JC. Identification of deleterious mutations within three human genomes. *Genome Res*. 2009;19(9):1553–1561.

17. Schwarz JM, et al. MutationTaster2: mutation prediction for the deep-sequencing age. *Nat Methods*. 2014;11(4):361–362.

18. Reva B, Antipin Y, Sander C. Predicting the functional impact of protein mutations: application to cancer genomics. *Nucleic Acids Res*. 2011;39(17):e118.

19. Shihab HA, et al. Predicting the functional consequences of cancer-associated amino acid substitutions. *Bioinformatics*. 2013;29(12):1504–1510.

20. Rentzsch P, et al. CADD: predicting the deleteriousness of variants throughout the human genome. *Nucleic Acids Res*. 2019;47(D1):D886–D894.

21. Yeo G, Burge CB. Maximum entropy modeling of short sequence motifs with applications to RNA splicing signals. *J Comput Biol*. 2004;11(2–3):377–394.

22. Coppe A, et al. Genomic landscape characterization of large granular lymphocyte leukemia with a systems genetics approach. *Leukemia*. 2017;31(5):1243–1246.

23. Cheon H, et al. Genomic landscape of TCRαβ and TCRγδ T-large granular lymphocyte leukemia. *Blood*. 2022;139(20):3058–3072.

24. Sandell RF, Boddicker RL, Feldman AL. Genetic Landscape and Classification of Peripheral T Cell Lymphomas. *Curr Oncol Rep*. 2017;19(4):28.

25. da Silva Almeida AC, et al. The mutational landscape of cutaneous T cell lymphoma and Sézary syndrome. *Nat Genet*. 2015;47(12):1465–1470.

26. Pizzi M, Margolskee E, Inghirami G. Pathogenesis of Peripheral T Cell Lymphoma. *Annu Rev Pathol*. 2018;13:293–320.

27. Niroula A, et al. Distinction of lymphoid and myeloid clonal hematopoiesis. *Nat Med*. 2021;27(11):1921–1927.

28. Talevich E, et al. CNVkit: Genome-Wide Copy Number Detection and Visualization from Targeted DNA Sequencing. *PLoS Comput Biol*. 2016;12(4):e1004873.

29. MacDonald JR, et al. The Database of Genomic Variants: a curated collection of structural variation in the human genome. *Nucleic Acids Res*. 2014;42(Database issue):D986-992.

30. Firth HV, et al. DECIPHER: Database of Chromosomal Imbalance and Phenotype in Humans Using Ensembl Resources. *Am J Hum Genet*. 2009;84(4):524–533.

31. Denny JC, et al. The “All of Us” Research Program. *N Engl J Med*. 2019;381(7):668–676.

32. Staels F, et al. Monogenic Adult-Onset Inborn Errors of Immunity. *Front Immunol*. 2021;12:753978.

33. Robins HS, et al. Comprehensive assessment of T-cell receptor beta-chain diversity in alphabeta T cells. *Blood*. 2009;114(19):4099–4107.

34. Pagliuca S, et al. Clinical and basic implications of dynamic T cell receptor clonotyping in hematopoietic cell transplantation. *JCI Insight*. 2021;6(13). https://doi.org/10.1172/jci.insight.149080.

35. Emerson RO, et al. Immunosequencing identifies signatures of cytomegalovirus exposure history and HLA-mediated effects on the T cell repertoire. *Nat Genet*. 2017;49(5):659–665.

36. Dean J, et al. Annotation of pseudogenic gene segments by massively parallel sequencing of rearranged lymphocyte receptor loci. *Genome Med*. 2015;7:123.

37. Huuhtanen J, et al. Single-cell characterization of leukemic and non-leukemic immune repertoires in CD8(+) T-cell large granular lymphocytic leukemia. *Nat Commun*. 2022;13(1):1981.

38. Lopez R, et al. Deep generative modeling for single-cell transcriptomics. *Nat Methods*. 2018;15(12):1053–1058.

39. Stuart T, et al. Comprehensive Integration of Single-Cell Data. *Cell*. 2019;177(7):1888-1902.e21.

40. Tsherniak A, et al. Defining a Cancer Dependency Map. *Cell*. 2017;170(3):564-576.e16.

41. Robinson MD, McCarthy DJ, Smyth GK. edgeR: a Bioconductor package for differential expression analysis of digital gene expression data. *Bioinformatics*. 2010;26(1):139–140.

42. Soneson C, Robinson MD. Bias, robustness and scalability in single-cell differential expression analysis. *Nat Methods*. 2018;15(4):255–261.

43. Love MI, Huber W, Anders S. Moderated estimation of fold change and dispersion for RNA-seq data with DESeq2. *Genome Biol*. 2014;15(12):550.

44. Yu G, et al. clusterProfiler: an R package for comparing biological themes among gene clusters. *OMICS*. 2012;16(5):284–287.

45. Tirosh I, et al. Dissecting the multicellular ecosystem of metastatic melanoma by single-cell RNA-seq. *Science*. 2016;352(6282):189–196.

46. Subramanian A, et al. Gene set enrichment analysis: a knowledge-based approach for interpreting genome-wide expression profiles. *Proc Natl Acad Sci U S A*. 2005;102(43):15545–15550.

47. Warde-Farley D, et al. The GeneMANIA prediction server: biological network integration for gene prioritization and predicting gene function. *Nucleic Acids Res*. 2010;38(Web Server issue):W214-220.

48. Shannon P, et al. Cytoscape: a software environment for integrated models of biomolecular interaction networks. *Genome Res*. 2003;13(11):2498–2504.

49. R Core Team. R: A Language and Environment for Statistical Computing. 2018. https://www.R-project.org/.
